# 3D electron microscopy and volume-based bouton sorting reveal the selectivity of inputs onto geniculate relay cell and interneuron dendrite segments

**DOI:** 10.3389/fnana.2023.1150747

**Published:** 2023-03-17

**Authors:** Erin E. Maher, Alex C. Briegel, Shahrozia Imtiaz, Michael A. Fox, Hudson Golino, Alev Erisir

**Affiliations:** ^1^Department of Psychology, University of Virginia, Charlottesville, VA, United States; ^2^School of Neuroscience, Virginia Tech, Blacksburg, VA, United States; ^3^Fralin Biomedical Research Institute, Roanoke, VA, United States

**Keywords:** SBEM, LGN, visual thalamus, unbiased sampling, retinogeniculate, corticogeniculate

## Abstract

**Introduction:**

The visual signals evoked at the retinal ganglion cells are modified and modulated by various synaptic inputs that impinge on lateral geniculate nucleus cells before they are sent to the cortex. The selectivity of geniculate inputs for clustering or forming microcircuits on discrete dendritic segments of geniculate cell types may provide the structural basis for network properties of the geniculate circuitry and differential signal processing through the parallel pathways of vision. In our study, we aimed to reveal the patterns of input selectivity on morphologically discernable relay cell types and interneurons in the mouse lateral geniculate nucleus.

**Methods:**

We used two sets of Scanning Blockface Electron Microscopy (SBEM) image stacks and Reconstruct software to manually reconstruct of terminal boutons and dendrite segments. First, using an unbiased terminal sampling (UTS) approach and statistical modeling, we identified the criteria for volume-based sorting of geniculate boutons into their putative origins. Geniculate terminal boutons that were sorted in retinal and non-retinal categories based on previously described mitochondrial morphology, could further be sorted into multiple subpopulations based on their bouton volume distributions. Terminals deemed non-retinal based on the morphological criteria consisted of five distinct subpopulations, including small-sized putative corticothalamic and cholinergic boutons, two medium-sized putative GABAergic inputs, and a large-sized bouton type that contains dark mitochondria. Retinal terminals also consisted of four distinct subpopulations. The cutoff criteria for these subpopulations were then applied to datasets of terminals that synapse on reconstructed dendrite segments of relay cells or interneurons.

**Results:**

Using a network analysis approach, we found an almost complete segregation of retinal and cortical terminals on putative X-type cell dendrite segments characterized by grape-like appendages and triads. On these cells, interneuron appendages intermingle with retinal and other medium size terminals to form triads within glomeruli. In contrast, a second, presumed Y-type cell displayed dendrodendritic puncta adherentia and received all terminal types without a selectivity for synapse location; these were not engaged in triads. Furthermore, the contribution of retinal and cortical synapses received by X-, Y- and interneuron dendrites differed such that over 60% of inputs to interneuron dendrites were from the retina, as opposed to 20% and 7% to X- and Y-type cells, respectively.

**Conclusion:**

The results underlie differences in network properties of synaptic inputs from distinct origins on geniculate cell types.

## 1. Introduction

In the visual system, several types of excitatory, inhibitory, and modulatory synapses from distinct origins converge and interact on the dendritic arbors of lateral geniculate nucleus (LGN) relay cells that represent different parallel pathways, and thereby modify or modulate visual signals before they are relayed to the cortex (McCormick and Prince, 1987; Erişir et al., 1997a,b; Sherman and Guillery, 1998; Jones, 2002; Rovó et al., 2012; Crandall and Cox, 2013; Bickford, 2019). The patterns of these synaptic interactions on individual cell types are likely to be specific, contributing to the functional distinctions within the neuronal circuits and pathways they represent. For example, glomerulus, a glia-ensheathed cluster of synaptic boutons from some, but not all types of geniculate input axons, are described as selective to X-type relay cells (Sherman and Guillery, 1996; Datskovskaia et al., 2001; Dankowski and Bickford, 2003). Similarly, while retinal synapses are mostly found on large-caliber, presumed proximal dendrites, corticothalamic synapses are mostly on thin-caliber, distal dendrites, suggesting spatial segregation of feedforward and feedback excitation on individual cells (Wilson et al., 1984; Wilson, 1989). Furthermore, relative numbers of individual inputs that converge on relay cell and interneuron dendrites may underlie the functional phenotypes of geniculate cells (Wilson et al., 1984; Montero, 1991). A key effort for identifying the selectivity of inputs from distinct origins for geniculate cell types and the patterns of co-innervation on dendritic arbors has been the use of transmission electron microscopy (TEM) combined with tract-tracing and immunolabeling of distinct synaptic terminals (Peters and Palay, 1966; Guillery, 1970; Ohara et al., 1989; Erişir et al., 1997b; Van Horn et al., 2000; Wang et al., 2001). More recently, the development of serial imaging approaches has provided opportunities for the characterization of input terminals using 3D reconstructions of axons and postsynaptic dendrites, convergence and divergence of inputs on geniculate cell types, and the motifs of synaptic connectivity that can contribute to the modification and modulation of visual signal (Denk and Horstmann, 2004; Helmstaedter et al., 2008; Hammer et al., 2015; Morgan et al., 2016; Morgan and Lichtman, 2020). While the serial blockface electron microscopy (SBEM) approach is not fully compatible for the immuno-identification of axon origins (however, see Boey et al., 2019 or Luján et al., 2021, for focused ion beam milling and scanning electron microscopy, FIB/SEM applications with immuno-labeled tissue), 3D reconstruction morphometry approach is particularly advantageous over 2D EM because the latter can be confounded by measurement and sampling errors. For example, while terminal cross-section area measurements from 2D sections provide simple estimates for cross-sectioned bouton size, the volume reconstructions from 3D stacks yield a direct measure for each bouton included in the dataset. Similarly, 3D image stacks are more suitable for confirming morphological features used as sampling criteria in quantitative EM analyses, including synapse structure and mitochondria contrast, as well as special circuitry features such as dendritic appendages with pre and postsynaptic zones. Finally, 3D EM has the advantage of revealing where the synapses are formed on the dendritic arbor along with the complement of other inputs that would contribute to local and global signal summation.

In the current study, we took advantage of the serial imaging technology and known morphological and morphometric properties of geniculate input terminals and aimed to determine if there was a specific organization of synaptic inputs from different origins onto relay cell dendrites. We also addressed if the variability in target selectivity of geniculate inputs correlates with any discernible variability in relay dendrite morphology that may hint at different synaptic circuitry properties governing distinct parallel pathways. To answer these questions, we reconstructed dendrite segments and terminal boutons in the binocular core region of the mouse LGN, which contains X- and Y-type relay cells (Guido, 2018). For volume-based sorting of geniculate terminals, we first developed an unbiased sampling approach to obtain volume measurements of synaptic boutons. This displayed a wide and multimodal distribution, indicating possible composition with multiple terminal subpopulations with unique morphologies and distinct origins. We then used statistical modeling to identify the volume cutoff criteria for these subpopulations, which are subsequently applied on a dataset of boutons that synapse on reconstructed dendrite segments. The selectivity of each bouton subpopulation for cell-type identified dendrite segments and their interactions with each other were analyzed using statistical comparisons and network analysis approaches. The results reveal novel evidence for the presence of at least four distinct retinal ganglion cell inputs in the geniculate nucleus and the selectivity of distinct inputs for specific compartments of putative X-type relay cell dendrites but not on Y-type relay cells. Furthermore, the contribution of putative retinal, inhibitory, modulatory brainstem and corticothalamic inputs on two types of relay cells and interneurons differ significantly, revealing the anatomical bases for pathway-specific processing and local inhibition in the LGN.

## 2. Materials and methods

### 2.1. Tissue preparation

The brain tissue for generating SBEM image stacks was prepared at Virginia Tech, Carilion Institute, and sent to Renovo Neural, Inc (Cleveland, OH) for imaging. Two adult C57 mice were deeply anesthetized and perfused transcardially with phosphate-buffered saline (PBS; 0.1MPB, 0.9% NaCl) followed by a mixture of 4% paraformaldehyde and 2% glutaraldehyde made in 0.1M cacodylate buffer. Brains were removed from the skull and cut coronally at 300 μm sections on a vibratome. The LGN was dissected from the appropriate sections and sent to Renovo Neural Inc for staining, embedding, sectioning, and imaging. The data for the current analysis are collected from two LGN stacks of 300 and 200 images that are 41 μm × 41 μm in dimensions (pixel size: 5 × 5 nm) and 75 nm apart. All animal procedures were approved by Virginia Tech IACUC. SBEM stacks used in this study are uploaded to BossDM.org depository.

### 2.2. SBEM image tracing

*Cell Bodies and Dendrites:* All image tracing was done using Reconstruct software [Synapse Web Reconstruct; RRID:SCR_002716; (Fiala, 2005)]. When a neuronal nucleus and somatic organelles (i.e., Golgi apparatus, rough ER) were discernable in image stacks, its cell body was traced in subsequent images to find the dendrite emergence points. At the dendrite emergence points, part of the soma tapered to a cylindrical form to form a primary dendrite. Secondary and tertiary branches were identified when a dendrite split into two thinner branches or gave off a side branch thinner than the parent dendrite segment from which it emerged. Additional dendrites that were not emanating from a soma within the stack were also reconstructed. Dendrite segments that displayed somatic organelles were identified as primary branches. Some, but not all, of the dendrite segments displayed thin filopodia that extended short distances from the dendrite shaft. Similarly, grape-like appendages (multiple, large spine heads, emanating from a thin stalk) were traced when encountered. The filopodia and grape-like appendages were classified along with the branching order of the dendrite shaft they emerged from. Dendrites that displayed F2 morphology (a collection of vesicles or presynaptic zone) were classified as interneuron dendrites; dendrites that lacked F2 morphology were classified as relay dendrites. All dendrite segments and cell bodies were traced until the profile of the traced object was no longer visible in the stack (i.e., extended outside the volume of tissue represented in the stack). Dendrite lengths and volumes were measured using the Z-length and 3D volume tools of Reconstruct. With the assumption that dendrites are roughly cylindrical structures, the dendrite caliber (diameter) was estimated with the formula: *d* = 2*√(volume ÷ (length × π)).

*Synapses and terminals:* To identify terminals synapsing on the reconstructed relay cell dendrites, first, all synaptic zones that appeared on each dendrite segment were traced and reconstructed. Next, the terminal boutons that form each synapse were traced and reconstructed. Extra care was taken to capture the entire presynaptic bouton and exclude the thinner inter-bouton axon segments devoid of vesicles from the reconstructions. Terminal volumes were computed using Reconstruct software.

*Morphology-based identification of terminal types and other structures:* Early TEM studies of the mammalian LGN described and classified synaptic terminals based on several morphological criteria [(Guillery, 1969a); also see Bickford (2019) for a review]. According to that terminology, a population of terminals is classified as RLP, owing to their round vesicles, relatively large cross-section areas, and pale (or lighter contrast) mitochondria. That the RLP terminals originate exclusively from retinal ganglion cells was demonstrated with enucleation studies (Szentágothai et al., 1966; Novotny, 1979). A more common terminal morphology was classified as RSD owing to the round vesicles, small cross-section area, and dark mitochondria Guillery, 1969a). Among the origins of the RSD terminals, corticothalamic (Jones and Powell, 1969) and cholinergic brainstem axons (Fitzpatrick et al., 1989) were most frequent. Within the RSD population, the brainstem cholinergic terminals are shown to be slightly larger than corticothalamic terminals, and each constituted about half of the RDS terminals (Erişir et al., 1997b). Guillery (1969a) also defined a third general class of terminal morphology, F, F1, and F2, based on the flattened or pleomorphic appearance of vesicles. The tract-tracing and GABA-immunolabeling studies confirmed that all F-type terminals originated from inhibitory neurons, including geniculate interneurons and the projection neurons from the thalamic reticular nucleus and the pretectum (Montero, 1986; Wang et al., 2001, 2002). Furthermore, the F2 type, which displays both presynaptic and postsynaptic properties, was confirmed to be vesicle-filled appendages emanating from GABAergic geniculate interneuron dendrites (Hamos et al., 1985; Montero, 1986). Along with geniculate relay cell dendrites and retinal RLP terminals, the F2-type terminals constitute three components geniculate triads, a glia-encapsulated zone involving an RLP terminal contacting a relay dendrite and an F2 profile, which in turn synapse on the same relay dendrite (Rapisardi and Miles, 1984; Hamos et al., 1985).

In our study, while we followed all definitions and conventions mentioned above, we have made several alterations in identifying criteria for terminal boutons in the 3D material. First, because our classification scheme would rely on bouton size, whether a bouton appears qualitatively as small or large could not have been a criterion. Similarly, pleomorphic vesicle morphology, the primary criterion to identify F-type terminals in the lack of GABA labeling, was not evident in SBEM images. On the other hand, we could reliably apply the qualitative criterion for mitochondria attribute used by TEM studies in the SBEM material, especially because examining the same bouton in consecutive sections provided additional confirmation for whether its mitochondria had a light (or pale) or dark appearance. As such, we sorted reconstructed terminal boutons based on the mitochondria contrast: Light Mitochondria (LM) and Dark Mitochondria (DM) boutons. Because many small boutons contained no mitochondria, these were classified as the No Mitochondria (NM) group. For some of our analyses, boutons containing dark mitochondria and those that do contain any within the reconstructed volume were combined in DNM (dark or no mitochondria) group because all NM boutons were connected to an axon with dark mitochondria. While we noted any bouton that displayed the F2 characteristics, that is, it had both a presynaptic and postsynaptic zone, no further criteria could be applied to differentiate F-type terminals from other DM terminals. In addition, because F2 boutons emanate from interneurons and relay cell dendrites are not ever presynaptic, we classified the dendrite segments that contain an F2 bouton or form a synapse on any profile anywhere along its reconstructed object an interneuron dendrite. The dendrite segments without any presynaptic zone were classified as relay cell dendrites. We noted the presence of a glomerulus by the ensheathment of multiple terminals and dendrites by glial processes. The synapses were identified by the parallel arrangement of the presynaptic and postsynaptic membranes at the synaptic cleft and the presence of docked vesicles on at least two adjacent sections. Asymmetric and symmetric synapse classification that is used in TEM for sorting synapses with thick postsynaptic density (PSD) as presumed excitatory and those with no discernable postsynaptic thickness as inhibitory, was not applied in the current SBEM study due to insufficient differentiation and resolution of PSDs.

### 2.3. Unbiased terminal sampling

**FIGURE 1:**
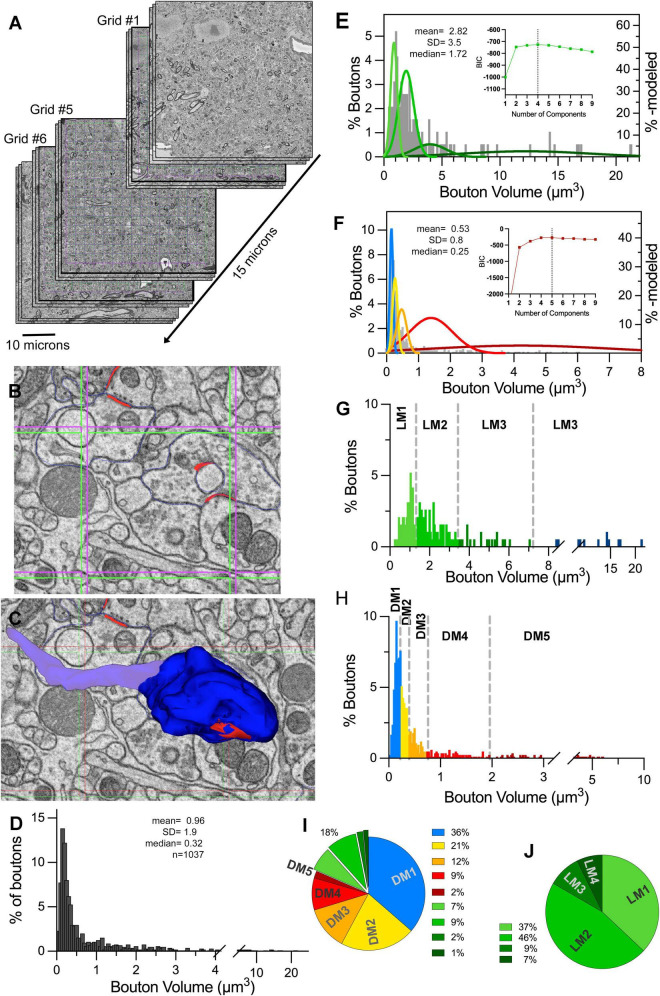
Determining the cutoffs for volume-based terminal sorting using unbiased terminal sampling (UTS) methodology and statistical modeling. **(A)** The scheme of placing stereological grids on an SBEM stack, yielding 1,364 sampling locations within a 40 μm × 40 μm × 15 μm tissue. For illustration purposes, only representative sections are depicted; and the Z-axis is expanded. **(B)** Within each stereological grid, synapses (outlined in red) are marked, including those touching the inclusion line (green lines) but excluding those touching the exclusion lines (purple lines). **(C)** The terminal boutons (e.g., dark blue) that form the “marked” synapses are reconstructed to reveal the 3D structure, and its volume is included in the UTS dataset. Care taken to not include the axon stalks or shafts (light blue) in the volume measurements. **(D)** The volume frequency histogram of all terminals collected with UTS, displaying a wide and irregular distribution. **(E)** The volume frequency distribution of boutons with light mitochondria (LM) terminals (gray bars) with overlaid normal distributions of four subpopulations modeled via BIC (four shades of green bell curves). Inset shows the BIC for models with different numbers of components. The vertical line marks the model with the highest BIC number. **(F)** Volume frequency distribution of boutons with dark or no mitochondria (DNM) terminals (gray bars) with overlaid normal distributions of five subpopulations modeled via BIC (blue, yellow, orange red, and burgundy bell curves). Inset shows the BIC for models with different numbers of components. The vertical line marks the model with the highest BIC number. **(G,H)** The cutoff values (vertical dashed lines) that are estimated via Monte Carlo simulations are applied to LM **(G)** and DNM **(H)** populations that are sampled via UTS. LM1, LM2, L3, and LM4 subpopulations in panel **(G)**, and DM1, DM2, DM3, DM4, and DM5 subpopulations in panel **(H)** are further color-coded. **(I,J)** The percent contributions of each subpopulation within the overall dataset **(I)** and within the LM population only **(J)**. Subpopulation color-coding for LM1-4 and DM1-5 correspond to those in panels **(G,H)**.

To collect a random sample of LGN terminals, six 15 × 15 stereology grids were placed within one of the LGN stacks, yielding 1,350 sequentially numbered “locations” across six grids (Figure 1A). Stereology grids were 33 sections (that is, 2.47μm) apart. Each location was defined by two inclusion and two exclusion sides (green and purple lines, respectively, in Figures 1B, C), yielding a 2.5μm distance between any two adjacent green (or purple) lines. As such, the stereology grids generated a 3D array of 1,350 cubes (or *locations*, as referred to in this manuscript) with 2.5μm at X, Y and Z dimensions. A random number generator was used to select locations, which were examined for the presence of a synapse within the inclusion lines; if a synapse crossed the exclusion line, it was excluded from the analysis. Next, each included synapse was traced starting on the tissue section containing the grid section and continuing through adjacent sections until it disappeared. The terminal bouton that formed the traced synapse was traced and reconstructed. For boutons that entrapped or wrapped around a filopodium within its volume, the reconstruction excluded the volume of the filopodia. Terminal tracing was completed when the terminal bouton form was no longer discernible. Data collection from the stack continued until 50% of all stereology array locations (675 randomly selected locations out of the1,350 locations defined in the stereology array) were examined, and terminals within, if present, were reconstructed. The locations that were within a soma, or did not display a synapse, were also noted. This procedure was repeated a second time by placing six stereological grids on different sections on the same SBEM stack. The final datasets contained 543 and 505 reconstructed terminal boutons.

### 2.4. Statistical modeling and analyses

*Model-based classification*: The Gaussian Normal Mixture Modeling feature of the R Package (R Project for Statistical Computing; RRID:SCR_001905) *MClust*, which assumes that distinct subpopulations that make up a heterogeneous population are normally distributed, is used to determine the parameters of subpopulation clusters within the Unbiased Terminal Sampling (UTS) dataset based on terminal volume (Scrucca et al., 2016). The selection of a model is based on the highest Bayesian Information Criteria (BIC) between models of different cluster numbers. The MClust package then yields each cluster’s sample size, mean, and standard deviation, which are used to construct distribution curves of model subpopulations.

*Monte Carlo simulation*: To determine a volume cut-off value between each pair of neighboring subpopulations, a Monte Carlo simulation was run for 10,000 iterations using the subpopulation parameters (mean, SD, and relative size) from the MClust analysis on the unbiased terminal sampling dataset. The simulated populations had 5X the number of observed subpopulation cluster sizes, while the relative subpopulation sizes were kept constant. Theoretical cutoff values were calculated as the average cutoff volumes from each of the 10,000 simulations. These cutoff values were subsequently applied to the empirical datasets to classify morphologically distinct axon terminals.

*Association rule analysis:* To determine patterns of connectivity between input types, association rule learning analysis, which determines relationships between frequent items in a dataset, was applied using the R package, “*arules*” (Hahsler et al., 2005). In this analysis, the relationship between two items in a dataset, for example, A and B, is defined as an if, then probability so *if A, then B*, meaning that if *A* is present, then *B* is present. To determine associations between terminals synapsing on relay cell dendrites in the LGN, each dendrite segment was treated as its own dataset, and the terminals present on those segments were treated as items in that dataset. Because the *arules* analysis takes the occurrence of any terminal type into account, regardless of its frequency, only the terminal types with frequency within the 95% confidence interval for a given dendrite segment were included in the analysis, resulting in the exclusion of rare occurrences on segments receiving a large number of inputs. The association between two terminals on a single dendrite segment was described using the confidence and lift values. The *confidence* of an association is the probability of finding terminal B when terminal A is also present. The lift value is the ratio of the co-occurrence of two terminals on the same dendrite segment to the occurrence of each item individually if they were independent (Hahsler and Hornik, 2009). As such, the *lift* accounts for the popularity, and it can reveal negative associations: If *lift* is equal to 1, its co-occurrence with the second item is at chance. If *lift* is greater than 1, then the presence of one item positively impacts the presence of the other, and if it is less than 1, then the presence of an item negatively affects the presence of the other. Heat map graphs of the *lift* associations were plotted using the *arules* package in R and Prism.

*Network modeling:* The R package “qgraph” is used to create a network model of the relationships between terminal clusters based on the confidence values of terminal associations (Epskamp et al., 2012). Networks are comprised of nodes, which represent individual terminal types, and edges, which represent how strongly two terminal types occur together (Harary, 1969). The size of the node reveals the centrality of the terminal type in determining the network. The *outflow* measure of centrality is used to identify how much one node influences other nodes, expressed as *outstrength* values.

*Statistical Comparisons and Graphs:* All graphs, descriptive statistics, and comparative statistics (including Mann–Whitney U, Kruskal–Wallis and D’Agostino & Pearson tests) were completed using Prism software version 9 (Graphpad). All figures were created and annotated using Adobe Creative Cloud Photoshop software.

## 3. Results

### 3.1. Volume-based classification of terminals in an unbiased dataset

A total of 1,048 terminal boutons were reconstructed using the Unbiased Terminal Sampling approach (Figures 1A–C). The stereological grid size and placement parameters accounted for the size range of geniculate synapses and boutons, eliminating the possibility of oversampling any input type: most, if not all, synapses appeared within only one stereological grid inclusion lines, and no bouton extended through the z-planes of two consecutive grid placements. Similarly, the z-placement of the array did not impact the randomization of the sample: After running two iterations of stereological grid-array placement in the same SBEM stack and examining 50% of the grid locations in each array, we obtained two independent datasets of 540 and 508 boutons, respectively. Analysis of terminal volumes from the two grid placements revealed that these two datasets were not statistically different (Mann–Whitney U, *p* = 0.77), suggesting that the stereological array placement accounted for the potential modularity of large geniculate glomeruli. The frequency distribution of bouton volumes ranged between 0.03 and 21.3 μm^3^ and displayed multiple peaks (Figure 1D). The dataset failed both normality and lognormality tests (D’Agostino & Pearson tests at alpha = 0.05, *p* < 0.0001, for both normality and lognormality), suggesting population heterogeneity. The terminal size frequency distribution of the terminals with light mitochondria (i.e., LM population; Figure 1E) was statistically larger than the terminals that displayed dark or no mitochondria (i.e., DNM population; MWU, *p* < 0.0001; Figure 1D). The LM and DNM datasets also failed to pass both the normality and lognormality tests: null-hypothesis for normality at D’Agostino & Pearson tests yielded *p* < 0.0001 for both LM and DNM datasets; null-hypothesis for lognormality yielded *p* = 0.0017 for LM and *p* < 0.0001 for DNM datasets.

The BIC-based modeling (using *MClust* R-package) of the LM and DNM populations revealed evidence for 4 subpopulations of LM boutons and 5 subpopulations of DNM boutons (Figures 1E, F insets). Monte Carlo Simulation approach is used to generate simulated datasets of subpopulations defined by the parameters yielded from the MClust modeling of LM and DNM populations. These simulations were used for computing the cutoff values for terminal volumes for each pair of subpopulations with overlapping tails and yielded the following values: 1.31; 3.34; 7.22 μm^3^ for LM, and 0.22; 0.39; 0.75; 1.95 μm^3^ for DNM populations (Figures 1G, H). The subpopulations flanked by these cutoff points are referred to as LM1-4 and DM1-5 throughout this paper.

In order to reveal the percent contribution of each distinct terminal subpopulation to the geniculate circuitry, the cutoff values were applied to the actual LM and DNM subpopulations of the unbiased dataset, and their ratios among the full dataset were calculated (Figures 1G–J): First, about 18% of all inputs in the Unbiased Terminal Sampling dataset displayed light mitochondria (Figure 1I), thus of retinal origin, and this ratio is consistent with the general estimates of retinal inputs to geniculate laminae in the cat, tree shrew, monkey, rat and mouse (Colonnier and Guillery, 1964; Mantyh and Kemp, 1983; Dreher et al., 1985; Erişir et al., 1997a,b, 1998; Bickford et al., 2000, 2010; Çavdar et al., 2011; Balaram et al., 2015). Interestingly, the retinal terminals in our dataset are composed of four subpopulations, LM1-4, suggesting that axons from different retinal ganglion cell types may have distinct morphological properties. These subpopulations contribute to the geniculate circuitry at different strengths: The two smaller-sized retinal populations that contain terminals that are smaller than 3.4μm^3^, constitute a total of 83% of all retinal terminals, and they account for 6.8 and 8.6% (15.4% in combination) of all terminals in the LGN (Figures 1I, J). The two larger-sized retinal terminal subpopulations (larger than 3.4 and 7.2μm^3^) constitute 16.6% of LM terminals, accounting for only a total of 1.5% of synaptic terminals to the LGN.

The non-retinal, DNM, terminals were composed of 5 distinct subpopulations, DM1-5. The two smallest-sized groups of DNM terminals (also referred to as DM1 and DM2 subpopulations throughout this manuscript), which are smaller than 0.2 and 0.4 μm^3^, respectively, constitute 37% and 21% of all LGN terminals. As these ratios are consistent with the 2D estimates of corticothalamic and brainstem cholinergic inputs to LGN (Erişir et al., 1997b), we refer to DM1 and DM2 as presumed cortical and brainstem terminals. The next two larger subpopulations (i.e., DM3 and DM4) had cutoff points at 0.7 and 1.9μm^3^ and constituted 12 and 9% of all inputs to the LGN, respectively. These are assumed to be inhibitory inputs, including from geniculate interneurons (dendritic appendages and axons) and thalamic reticular nucleus (Campbell et al., 2020). The fifth subpopulation with dark mitochondria (i.e., DM5) is composed of a small number (2% of all boutons) of terminal boutons that were large (between 1.9 and 6.0 μm^3^). Such large boutons with dark mitochondria were also encountered in 2D TEM studies, and they have been classified as RLD terminals, based on their round vesicles, large cross-section size and dark mitochondria (Wilson et al., 1984; Hamos et al., 1985). While the origin of the RLD terminals is not yet known, it was speculated to be local axon collaterals of geniculocortical relay cells (Bickford et al., 2008).

### 3.2. Qualitative properties of relay cell dendrites

**FIGURE 2:**
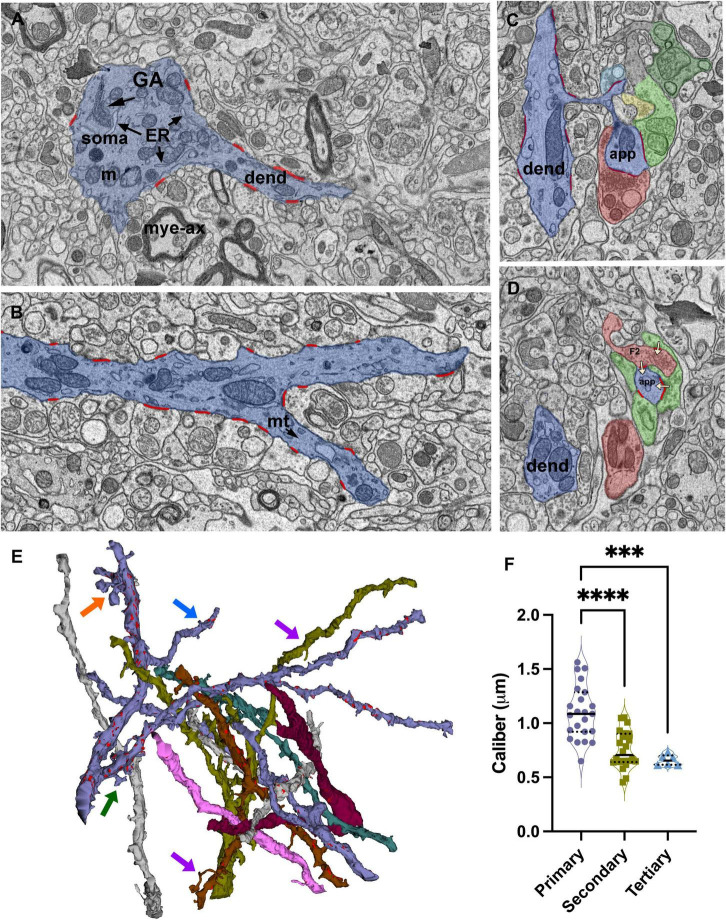
Identification and reconstruction of primary, secondary, and tertiary dendrite segments of relay cells. **(A)** The dendrite segments that emerge from profiles containing soma-specific organelles, including Golgi apparatus (GA) and endoplasmic reticulum (ER), are identified as primary segments. Mye-ax: myelinated axon; m: mitochondria; soma: cell body; dend: dendrite. **(B)** SBEM image of a primary dendrite (blue) splitting into two secondary branches. All synapses (red) formed on the reconstructed dendrite segments are identified and traced. mt: microtubules. **(C,D)** Cross-sections through a dendritic spine complex or a grape-like appendage (blue), which receives multiple synapses (traced in red; white arrows mark the direction of the synapses) from various input boutons; distinct boutons are pseudocolored following the color-coding scheme of volume sorting. Note that the appendage is engaged in a triad: a presynaptic bouton [F2; **(D)**] also receives a synapse from another bouton that contains light mitochondria and synapses on the same appendage. **(E)** 3D reconstructions of some of the dendrites in the relay dataset. Synapses are marked in red along representative dendrites. Most dendrites give off thin filopodia (e.g., blue arrow) that emerge close to a synapse on the dendrite shaft and protrude into the presynaptic bouton; the synaptic zone often extends along protruding filopodium. Other filopodia emerge from non-synaptic regions, and extend long distances in the extracellular matrix without receiving synapses (e.g., purple arrows). Spines emerge from the dendrite with a thin stalk and form an enlarged head that receives synapses from 2 to 3 different boutons (e.g., green arrow). A grape-like appendage is a spine complex where 2–3 spine heads emerge from the same thin stalk (e.g., orange arrow). Like single spines, grapes also receive multiple distinct synapses and engage in triads. **(F)** The size distribution of calibers of primary dendrites are significantly larger than both the secondary and tertiary dendrite segments (Kruskal–Wallis, Dunn’s multiple comparisons, ^****^ < 0.0001; ^***^ = 0.0004), Tertiary dendrite caliber distribution is statistically indistinguishable from secondary branches (*p* = 0.9230). Because the 75th percentile range of calibers in the tertiary branches group overlaps with the 50th percentile of the secondary branches group, no size cutoff can be used to categorize the branches by only a caliber criterion.

In order to identify and categorize the patterns of synaptic terminal boutons on geniculate relay cell dendrites, a total of 80 individual dendrite segments belonging to 21 distinct dendrites were reconstructed from two SBEM stacks (Figures 2A–E and Supplementary Figure 1). These were classified as relay dendrites because none of the segments or their parent dendrites displayed presynaptic zones, a morphological characteristic of interneurons (Figure 2D). The dendrite segments were categorized as primary, secondary, tertiary, or quaternary branching order sequentially from the primary segment that either emerged from a soma or contained somatic organelles (i.e., rough endoplasmic reticulum or Golgi apparatus- Figures 2A, B).

Dendrite segments, in general, either end by splitting into two “daughter” branches (Figure 2A) or give off a single side branch along their lengths. Some side branches are relatively thinner than the parent segment, and these extend out as a secondary (or tertiary segment, if they branch out of a secondary segment) branch; these receive synapses, and some give out branches themselves. When the dendrite segment split into two daughter segments, the thicknesses of the daughter segments are comparable to each other and thinner than the parent segment (Figures 2A, C).

Many other side branches are very thin and display filopodia morphology (Figure 2E, purple arrows). While most filopodia extend without any contact from terminals, some filopodia coil in on themselves and are trapped in large terminals, creating protrusions found in some large terminal boutons (Figure 2E, blue arrow). The synaptic zones of the large enveloping terminals often extend on these protruding filopodia, suggesting that postsynaptic filopodia may serve the function of increasing the active zone size of a synapse. In contrast, some filopodia extend away from the parent segment for relatively long distances without receiving any synapses. Filopodia are found emerging from primary, secondary, or tertiary segments.

In addition to filopodia, some segments display postsynaptic appendages classified as *spines* and *grapes.* Spines emerge from the dendrite shaft as a thin stalk and form a swelling or a mushroom top that receives 1–3 synapses (Figure 2E, green arrow). These are different than synapse-bearing filopodia protrusions in that protrusions and the parent dendrite are postsynaptic to the same bouton, whereas a spine receives a distinct synapse or, most often, multiple distinct synapses. A grape is a collection of 2–3 spines that emerge from a single stalk (Figure 2E, orange arrow; Supplementary Figure 2). Spines and grapes often emerge from secondary dendrites, and to a lesser extent, from primary dendrites and never from tertiary branches. A subset of dendrites reconstructed in the current dataset display spines or grapes; the spine- or grape-bearing dendrites give off many of these appendages.

**FIGURE 3:**
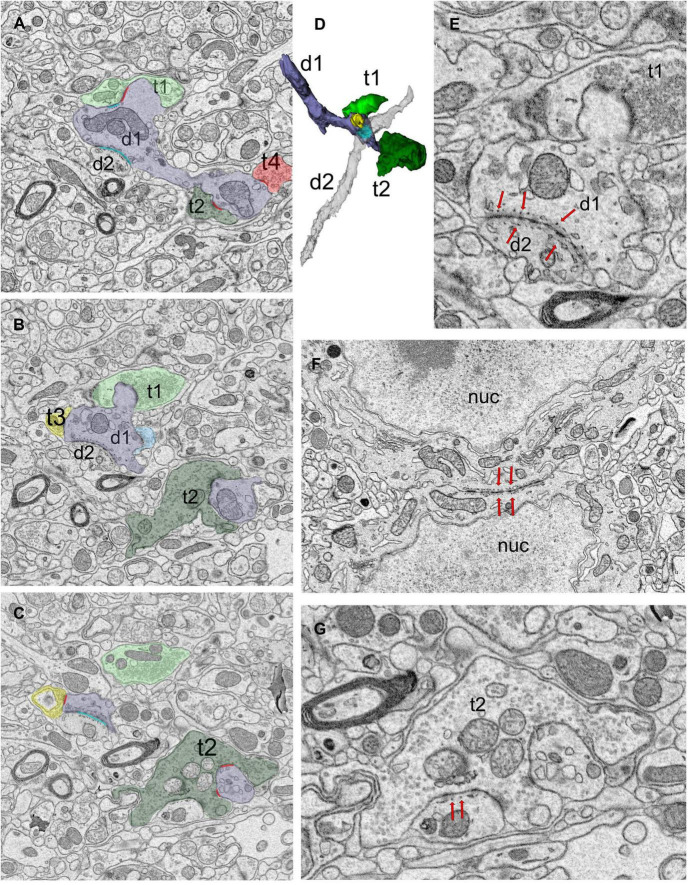
Dendro-dendritic puncta adherentia (PA) is a characteristic of a relay cell type. **(A–C)** Cross-section images of a reconstructed dendrite (d1, shaded lavender) that receives synapses from several boutons (t1-t4, pseudo-colored based on UTS volume-sorting classification). Synapses are marked in red **(A,C)**. The dendrite is also connected to a second dendrite (d2) via PA [marked in cyan in panels **(A,C)**]. **(D)** The reconstruction of the dendrites (d1 and d2, shaded lavender and gray, respectively) forming PA revealed they were both relay cell dendrites, and neither displayed any filopodia, spines or grape-like appendages. **(E)** Higher magnification view of the dendrodendritic PA (red arrows) reveals close membrane appositions between the plasma membranes and fine tubules or filaments at each side of the dendro-dendritic junction. **(F)** A PA structure between the neuronal somata (red arrows mark the structure from both sides). The dendrites of such somata that form somatosomatic PAs also displayed dendrodendritic PAs. nuc, nucleus. **(G)** PA or filamentous junctions (red arrows pointing to structure from the postsynaptic aspect) are frequently observed along with the synapses of large retinal terminals in the LGN. The retinal bouton cross-section (t2) with filamentous junction is from the same bouton labeled as t2 in panels **(A–C)**, and it synapses on d1 profile that is depicted in panels **(A–D)**. To note, d1 also forms a dendrodendritic PA with d2.

Puncta adherentia (PA), an ultrastructural feature characterized by tight appositions between the membranes of two neuronal elements, are encountered within our sample, occurring between two dendrites, two somata, or at the synaptic zone (Figure 3). In the case of dendrite-to-dendrite adherence contacts, both dendrites are from relay cells (Figures 3A–E). Interestingly, dendrodendritic adherent junctions and grape-like appendages never occur on the same dendrite segment or arbor. Furthermore, the dendrite segments that display dendrodendritic PA do not display grape-like appendages nor receive a synapse from an F2 terminal (a bouton postsynaptic to another bouton that is presynaptic to the same relay segment), within the reconstructed bouton volumes. Thus, relay cells that receive triadic synapses and those that form dendrodendritic adherent contacts with other relay dendrites may represent two distinct geniculate cell types. For the remainder of the analysis, we refer to the first type, that is, the dendrites that receive triadic synapses as belonging to X-like relay cells, based on the earlier findings revealing that F2 boutons and triads were associated with X-type relay cells and rarely, if at all, with Y-type relay cells (Wilson, 1989; Sherman, 2004). The dendrites that did not fit the morphological criteria for X-like relay cells (i.e., those not associated with triads) are classified as belonging to Y-like relay cells. It should also be noted that the current analysis now reveals a novel morphological feature, dendrodendritic PA, specific to the Y-like relay cells. In our reconstructed dendrites sample, 43 segments were from the triad-receiving cell type (X-like relay cell), and 37 were from the PA-forming cell type (Y-like relay cell).

### 3.3. Correlation of dendrite order and dendrite caliber

The calibers of dendrite segments in the dataset displayed a wide range (min: 0.43l max:2.27 μm; mean = 0.91 μm). When the dendrite order could be identified based on morphological criteria, the primary dendrite segments were thicker than both the secondary and tertiary segments (1.09+/−0.25 vs. 0.75+/−0.18 and 0.66+/−0.04 and 0.66 ± 0.04 μm, respectively; Figure 2F; Kruskal-Wallis ANOVA, *p* < 0001; see figure legends for pairwise *p* values). However, there was a considerable overlap among caliber sizes between primary and secondary branches, and more prominently between secondary and tertiary branches, which precluded the possibility of using caliber as a criterion to identify the order of any dendrite segment. That is, the caliber of a dendrite branch is not a good predictor for its branching order nor for the segment’s distance from the cell body. The calibers of dendrite segments belonging to triad-receiving vs. PA-forming cell types were not statistically different (MWU, *p* = 0.8).

### 3.4. Qualitative and quantitative properties of terminal boutons synapsing on relay cell dendrites

**FIGURE 4:**
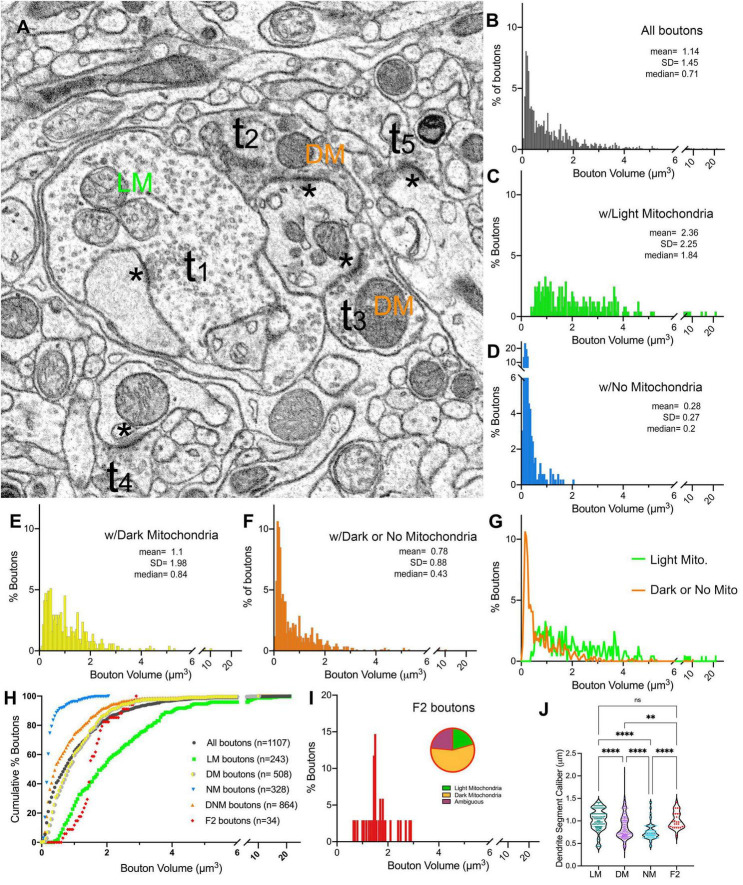
Terminal boutons that synapse on relay dendrites. **(A)** Reconstructed boutons are initially classified as belonging to light-mitochondria (LM; t1) or non-LM or DNM (i.e., those with DM or No mitochondria; t2-t3) populations. While smaller terminals may contain no mitochondrion within the cross-section (e.g., t4, t5) or the reconstructed bouton, when followed up through the image stacks, these almost always emerge from axons containing dark mitochondria. The symbol * marks the postsynaptic sites. **(B–F)** Volume distribution histogram of all boutons **(B)**, and boutons with light mitochondria **(C)**, with no mitochondria **(D)**, with dark mitochondria **(E)**, and with dark or no mitochondria **(F)**. Mean, SD and median values are in μm^3^. **(G)** The frequency distributions of larger-sized DNM and smaller-sized LM boutons overlap. **(H)** Cumulative percentage plot of all boutons, those with light mitochondria, dark mitochondria, no mitochondria, dark and no mitochondria combined, and the boutons that displayed pre- and postsynaptic morphology (i.e., F2 boutons). Except for LM vs. F2 boutons, all pairwise comparisons of distributions were statistically different (*p* < 0.0001; Kruskal–Wallis, followed by Dunn’s multiple comparisons test). **(I)** Volume distribution of F2 terminals reveals these overlap both with LM and DNM populations. Note that the mitochondria in about half of F2 boutons could not be unambiguously classified as dark, and some had light mitochondria (inset pie chart). **(J)** The comparison of dendrite caliber among dendrite segments that form synapses with each type of morphologically classified bouton (Kruskal–Wallis followed by Dunn’s multiple comparisons test). ^**^p = 0.0029; ^****^p < 0.0001.

In order to characterize synaptic patterns onto relay cell dendrites, all boutons synapsing onto the relay cell dendrites in our dataset were traced, the volumes were calculated, and the mitochondria within each bouton or its axon were categorized as “light” or “dark” (Figure 4A and Supplementary Figure 3). Terminal boutons synapsing on relay dendrites display a wide range of sizes (0.01 to 21.13 μm^3^; Figure 4B). The distribution is multimodal, consistent with the contribution of inputs from multiple origins, each with a unique bouton size phenotype. The multimodal distribution is also evident in subpopulations sorted by mitochondria hue (i.e., those that contain LM, DM, or no mitochondria) (Figures 4C–E). The boutons that do not contain a mitochondrion in their reconstructed volume are the smallest (range: 0.03 to 2.0 μm^3^), although the size distribution of this group overlapped with that of the group of boutons with dark mitochondria (range: 0.01 to 10.25 μm^3^), with over 85% of terminals with dark mitochondria smaller than the largest no-mitochondria group (Figures 4D, F). Furthermore, almost all no-mitochondria boutons displayed dark mitochondria just outside of the traced bouton. Therefore, we combined no- and dark mitochondria boutons in the same group of Dark/No Mitochondria (DNM) terminals, representing all non-retinal inputs. The distribution of volumes in LM and DNM groups were statistically different (K-W, Dunn’s adjusted *p* < 0.001) yet overlapping (Figures 4G, H). The boutons that displayed F2 morphology (i.e., displaying both a presynaptic and a postsynaptic zone) were found to have light, dark or ambiguously contrasted mitochondria (Figure 4I). The volume distribution of F2 boutons was not statistically different from those of LM and DNM terminals (*p* > 0.9), whereas F2 boutons were larger than the no-mitochondria group (*p* < 0.0001; Figure 4H). While the dendrite segment caliber contacted by LM and F2 boutons were similar, all other pairwise comparisons of dendrite caliber for LM, DM and NM boutons revealed these groups might have a preference for dendrite segments with certain caliber (Figure 4J).

**FIGURE 5:**
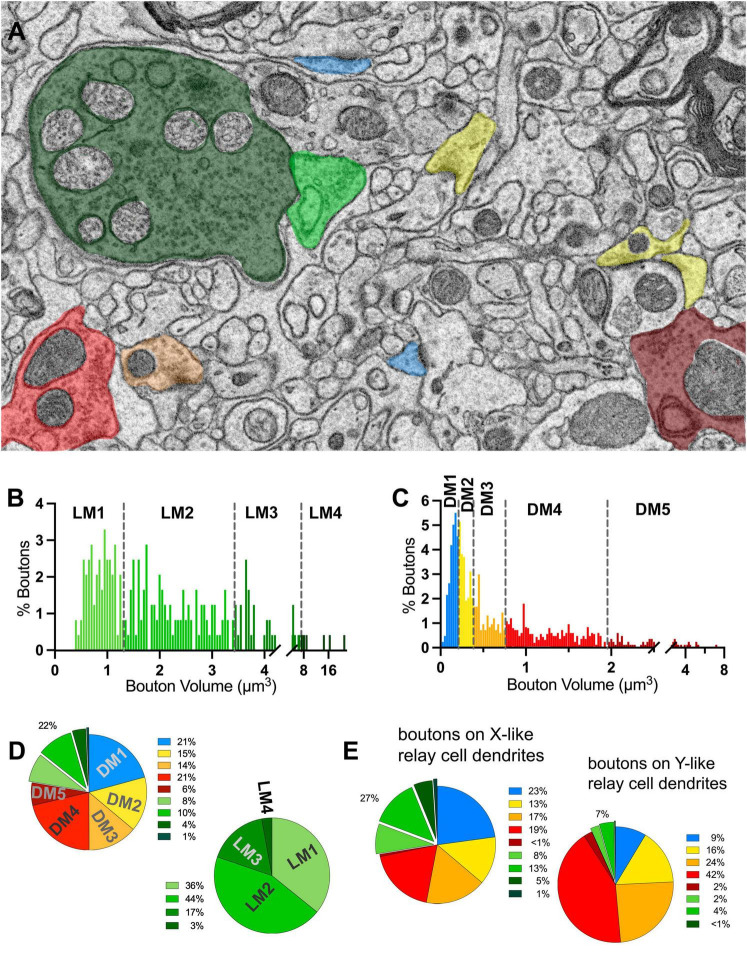
Volume sorting of boutons synapsing on relay dendrite segments. **(A)** The cutoff values obtained in the UTS analysis are applied to terminal boutons that synapse on relay cells. Boutons are pseudocolored for subpopulations, same as in Figure 1, except for mitochondria to illustrate the light- or dark-contrast morphological criteria used for initial sorting of LM and DM boutons. **(B,C)** The frequency distribution of LM **(B)** and DNM **(C)** boutons reveal multimodal distributions for each group sorted by the morphological criterion for mitochondria appearance. The UTS cutoff values (dashed lines) distinguished four subpopulations for LM and five subpopulations for DNM groups. **(D)** Contribution of nine subpopulations of boutons synapsing on relay dendrites. Retinal boutons constituted 22% of all synapses, while ∼40% of synapses (top pie chart) are from two subpopulations with the smallest bouton sizes (presumed cortical and brainstem inputs). Among the retinal boutons (bottom pie chart), the two largest-sized boutons constitute 20% of all retinal boutons and 5% of all boutons. **(E)** Dendrites that display spines or grape-like appendages (top pie chart) receive more frequent retinal boutons (27%) than the relay dendrites that display dendrodendritic PA (7%). The contribution of cortical and brainstem inputs on the dendrite segments with PA is also less prominent than on the relay dendrites with spines.

To classify terminals into putative origins, the cutoff values for terminal volumes derived from MClust analysis of the unbiased terminal dataset were applied to all terminals traced on relay cell dendrites (Figures 5A–C). LM terminals on relay dendrites comprised 22% of all terminals synapsing onto relay cell dendrites (Figure 5D). Of the LM terminals, 80% had the smallest two terminal volumes and 20% were those with the two larger terminal volumes (Figure 5B). Among the DNM terminals, the largest-sized subpopulation was the sparsest (8%; Figure 5D).

**FIGURE 6:**
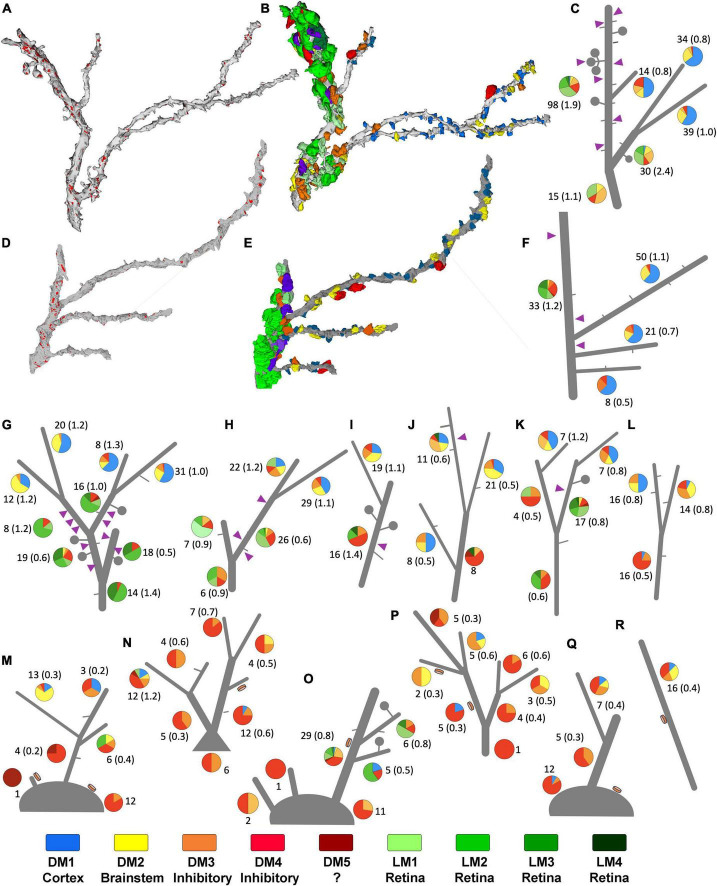
Sorting of relay dendrites based on exclusive morphological features. **(A–F)** Two examples of reconstructed relay dendrites and the distribution of boutons on distinct arbor segments. Synapses [red in panels **(A,D)**], and volume-sorted bouton subtypes **(B,E)** are illustrated on dendrite reconstruction (gray). The color scheme for volume-sorted boutons in panels **(B,E)** matches Figures 1, 5, and the legend at the bottom of this Supplementary Figure 2 boutons that partake in triads are colored purple regardless of their volume-sorted subpopulation. Stick figures of dendrites **(C,F)** are used to illustrate the contribution of each volume-sorted bouton subpopulation on distinct arbor segments (pie charts- legend at the end of the figure applies). The numbers under each pie chart indicate the number of boutons that synapse on a particular dendrite segment and the frequency of synapses (in parenthesis) along the length of that segment. Purple triangles indicate the approximate location of a triad. **(G–R)** While many reconstructed relay dendrites in our dataset displayed filopodia (short gray lines) and spines/grape-like appendages (lollipops), only a group received synapses from F2 type terminals (purple triangles) forming triads. **(G–K)** In contrast, others displayed dendrodendritic PA (orange disks) and sparse filopodia **(M–R)**. While processes that can be classified as spines were also occasionally encountered on this second group, no triads were found. The dendrites engaged in triads at any of their segments are categorized as X-type, and those display a PA are categorized as putative Y-type relay dendrites. Dendrite l was uncategorized for the network analysis.

When we further parsed the reconstructed dendrites into two morphologically distinct groups, one prominently engaged in triads (X-like cell; Figures 6G–K), the other bearing dendro-dendritic adherent contacts (Y-like cells; Figures 6M–R), the contribution of different bouton types to their circuitry revealed a specificity. For dendrites with triads, over a quarter of their inputs consisted of LM1-4, presumed retinal boutons, compared with only 7% for dendrites with PMichael A. Fox Furthermore, almost 70% of the inputs onto dendrites with PA were DM3-4 type, presumed inhibitory inputs from interneurons or the TRN, in contrast to the triad-bearing presumed X-like cell dendrites, which received about 36% of their inputs from DM3-4 boutons (Figure 5E).

### 3.5. Patterns of origin-specific terminals on relay dendrites

To study patterns of origin-specific terminals on LGN relay cell dendrites on visually displayed data, reconstructed terminal boutons were color-coded based on their mitochondria and volume-sorted subpopulations (Figure 6). As previously mentioned, dendrites with F2 innervation and glomeruli did not display PA and vice versa. This property was evident when all dendrites and their color-coded synaptic terminals were inspected visually (Figures 6G–R). In addition, dendrites with triads or F2 terminals (i.e., X-like relay cells) had more retinal and cortical inputs than the dendrites with PA (i.e., Y-like relay cells). Most strikingly, the retinal (i.e., LM1-4) and cortical (i.e., DM1) terminals were also largely segregated from each other (Figures 6G–L). While most putative retinal terminals were found on dendrites proximal to the soma, terminals providing putative feedback from the cortex were found on smaller, more distal dendrites (Figures 6G–L). In contrast, Y-like dendrites had fewer retinal and cortical terminals (Figures 6M–R).

### 3.6. Selectivity of origin-specific terminals on relay dendrite segments

**FIGURE 7:**
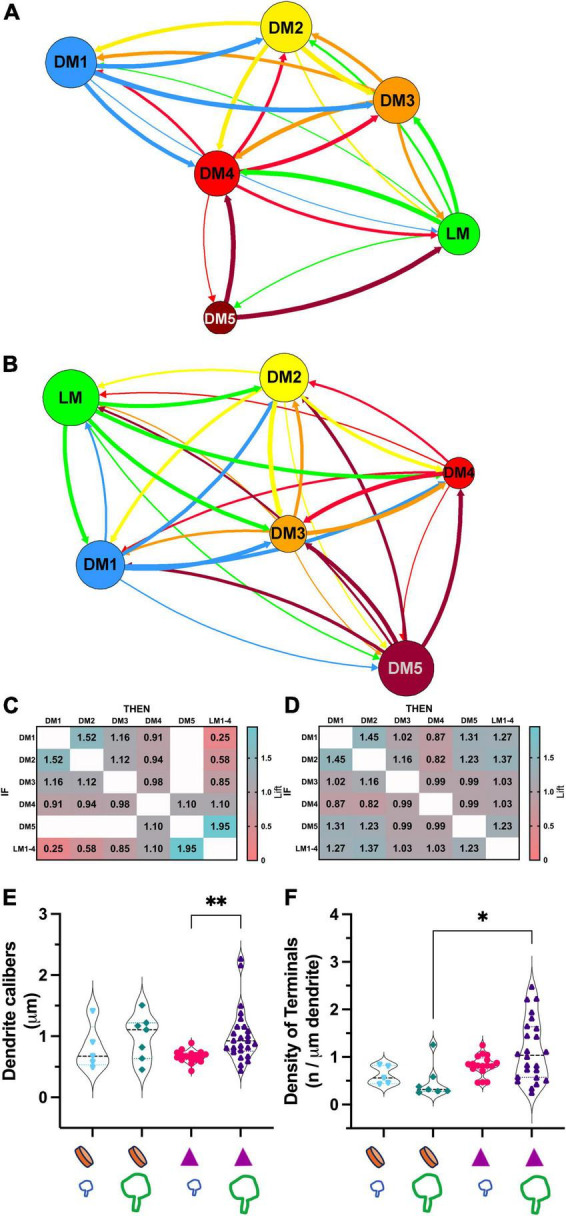
Association analysis reveals the segregation of retinal and corticothalamic boutons on relay dendrites that form triads. **(A)** The network analysis for outstrength associations among each bouton type that synapse on dendrite segments of X-type relay arbors. All retinal boutons (green- LM) are grouped; the bouton groups with dark or no mitochondria are marked following the same color scheme in Figure 5 (DM1-5). The line thickness indicates the strength of association from one terminal type to another type; arrows indicate the direction of the association. Corticothalamic (DM1, blue) and retinal (LM, green) boutons are strongly associated with all other bouton types but not with each other. **(B)** Network analysis for outstrength associations among each bouton type that synapse on dendrite segments of Y-type relay arbors. Retinal boutons are strongly associated with all other bouton types except for the largest DNM boutons (DM5). Corticothalamic boutons (DM1) are most strongly associated with DM2, DM3 and DM4 type boutons, putative GABAergic and brainstem cholinergic inputs. **(C)** Lift values from the association rules analysis for X-type relay dendrite segments. Lift values indicate the probability that the occurrence of a type of bouton (IF rows) influences the occurrence of another type (THEN columns) on the same dendrite segment. A lift value greater than 1 indicates that the occurrence of an “IF” bouton has a positive effect on the occurrence of the “THEN” bouton. A lift value near 1 indicates that the occurrence of the “IF” bouton has almost no effect on the occurrence of the “THEN” bouton type. A lift smaller than 1 indicates that the occurrence of the “IF” bouton type has a negative effect on the occurrence of the “THEN” bouton type. The strongest positive lift associations are found between relay boutons and the largest-sized boutons with dark mitochondria (lift = 1.95). Corticothalamic (DM1) and brainstem (DM2) boutons also influence each other’s occurrences positively (lift = 1.52). The strongest negative lift associations are found between retinal and corticothalamic terminals (lift = 0.25), indicating that these bouton types do not synapse on the same dendrite segments, that is, they are segregated on distinct branches. **(D)** Lift values from the association rules analysis for Y-like relay dendrite segments. All conventions are as described in panel **(C)**. No positive or negative lift associations exist between any two types of boutons, indicating that the input types on Y-like relay dendrite segments are not segregated. **(E)** The comparison of dendrite calibers for two relay cell types receiving corticothalamic and/or retinal synapses. The X-like dendrite segments (purple triangle symbol) that receive retinal synapses (large green bouton symbol) have larger calibers than those that receive corticothalamic synapses (small blue bouton symbol). *p* = 0.0002 with Mann–Whitney U two-tailed, and **p = 0.006 with Kruskal Wallis test with Dunn’s multiple comparisons (marked on graph). The dendrite caliber is not correlated with a corticothalamic or retinal synapse on Y-like dendrite segments (orange disk symbol on x-axis); *p* = 0.53. **(F)** The comparison of the frequency of synapses by corticothalamic (small blue bouton symbol on the x-axis) and by retinal (large green bouton symbol on the x-axis) on the dendrite segments of cell types that display a PA (orange disk on the x-axis) or triads (purple triangles on the x-axis). The retinal and corticothalamic bouton synapsing frequency was not statistically different for either cell type. Synapse frequency on X-type dendrites (with triads) is moderately higher than on Y-type dendrites (with PA) using Kruskal-Wallis, with Dunn’s multiple comparisons test (*p = 0.018, marked on graph), although more stringent Mann-Whitney test yields no significant differences (*p* = 0.089).

To address if certain inputs preferentially avoid forming synapses on a dendrite branch populated by another input type, we used a statistical modeling approach, association rules analysis. *ARules* analysis aimed to reveal the likelihood for two items to co-occur. In operational terms, we computed the confidence level for the likelihood for a terminal type B to synapse on a dendrite if a terminal type A synapsed on that same dendrite. Confidence values were used to calculate the outstrength for each terminal subpopulation. Outstrength reflects the influence of a given subpopulation over the presence of the other subpopulations (Figures 7A, B). The network analysis of dendrite segments bearing appendages (X-like) revealed that the retinal terminals (i.e., LM) had a strong influence for all terminals except for the corticothalamic terminals (i.e., DM1). In other words, the presence of a retinal terminal on a dendrite segment impacts (or predicts) the presence of all other types except for cortical terminals (Figure 7A). Similarly, the outstrength of corticothalamic boutons was the weakest for retinal terminals, suggesting that retinal and corticothalamic boutons do not co-occur on X-type cell branches. It is worth noting that, unlike DM1, the second smallest-sized (DM2), which is presumed brainstem boutons, did not have reciprocally weak outstrengths with retinal boutons. In contrast, the outstrengths of retinal (LM) and presumed corticothalamic boutons (DM1) with each other are moderate or high for Y-type relay cells (Figure 7B), thus suggesting that these two bouton types can occur together on the same dendrite segments. Furthermore, the comparison of lift values for each pairwise association rules within X-type cells revealed that the presence of a corticothalamic or a retinal terminal on a dendrite segment rendered the occurrence of a retinal or corticothalamic terminal, respectively, on the same dendrite segment highly unlikely (Figure 7C). A similar selectivity was not apparent for dendrites bearing dendrodendritic PA (putative Y-type; Figure 7D). These findings suggest that branch-selective segregation of retinal and cortical inputs is a property of X-type relay cells only.

Other differences between the connectivity patterns of retinal and corticothalamic (DM1) terminals on X- and Y-type relay cells were noted: The caliber of Y-type relay dendrites contacted by retinal terminals were significantly thinner than those of X-type ones (0.66 ± 0.4 vs.1.02 ± 0.4μm mean ± SD; Kruskal Wallis test, Dunn’s adjusted *p* = 0.006; Figure 7E). In contrast, no statistical difference was found between the calibers of Y-type dendrites that receive retinal or cortical inputs (Figure 7E), providing additional evidence that retinal and cortical boutons are not segregated across thicker proximal and thinner distal dendrites of Y-type relay cells. Similarly, the density of retinal terminals on X-cell dendrite segments was somewhat higher than those on Y-cell dendrites (1.14 ± 0.6 vs. 0.47 ± 0.4μm mean ± SD; Kruskal Wallis test, Dunn’s adjusted *p* = 0.018, although more stringent Mann-Whitney test yields no significant differences; *p* = 0.089; Figure 7F), while corticothalamic synapse density did not differ from that of the retinal synapses on X- or Y-type cells, nor between two cell types (Figure 7F). These findings may suggest fundamental differences in which X-type and Y-type relay cells integrate sensory and corticothalamic excitation.

### 3.7. Selectivity of inputs on interneurons

**FIGURE 8:**
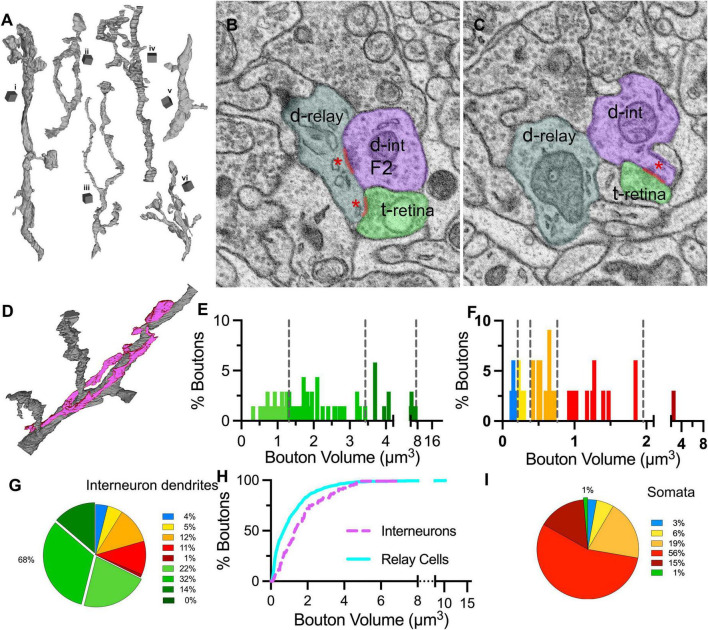
Distribution of bouton types on interneuron dendrites and somata. **(A)** 3D reconstructions of six **(i–vi)** interneuron dendrite segments. Cubical scale bar represents 1 μm ×1 μm. Interneuron dendrites have varying calibers and display many varicosities and appendages. **(B,C)** SBEM images across the two near-adjacent cross-sections of an interneuron dendrite (d-int/F2; pseudocolored in purple), a retinal bouton (t-retina, light green) and a relay dendrite (d-relay; blueish gray). Three synapses of the triad are marked with a red line and a red asterisk at the postsynaptic sites. **(D)** Interneuron dendrite segments (purple) often traveled along a relay dendrite (gray) and engaged in multiple triadic arrangements. **(E)** The cutoff values obtained in the unbiased terminal sampling are used for volume-sorting of distinct retinal terminals synapsing on interneurons. The largest-sized retinal terminals are not represented in this dataset. **(F)** The cutoff values obtained in the unbiased terminal sampling is used for volume-sorting of distinct non-retinal terminal subpopulations synapsing on interneurons. Two medium-sized, presumed inhibitory subpopulations represent the majority of non-retinal inputs. **(G)** The relative contributions of nine bouton subpopulations as synaptic inputs to interneuron dendrites. Retinal boutons provide close to 70% of inputs to interneurons, whereas corticothalamic and brainstem inputs constitute fewer than 10% of synapses. **(H)** Cumulative frequency distribution of bouton volumes on relay (cyan line) and interneuron (purple dashed line) dendrites. As the smallest-sized corticothalamic and brainstem inputs provide the largest portions of inputs of relay dendrites, the interneuron dendrites receive larger-sized boutons in general. **(I)** The relative contributions of nine bouton subpopulations as synaptic inputs onto neuronal cell bodies. By far the largest input on somata comes from inhibitory boutons, whereas retinal and corticothalamic boutons are rare.

To reveal if the contribution of synaptic inputs on interneuron dendrites mirrors that on relay cells, seven dendrite segments that displayed at least one presynaptic specialization (i.e., a cluster of vesicles were located by the dendrite membrane and at least one vesicle was “docked”, that is touching the membrane) and the synaptic boutons that were presynaptic to the dendrite were reconstructed (*n* = 103; Figure 8A). The presynaptic sites formed by the reconstructed dendrite (i.e., the F2 synapses), were also marked (Figures 8B–D). In contrast to relay dendrites, the interneuron dendrites had a tortuous and bulbous appearance, displayed many swellings, and gave appendages, where a large swelling was attached to the dendrite shaft with a thin stalk (Figure 8A, segment *i*). The caliber of the dendrite varied, and the branching was not associated with any change in the daughter dendrite’s caliber. The synapses on interneuron dendrites were primarily clustered around the varicosities and appendages, leaving large segments that are only surrounded by a glial sheet that isolated the dendrite from other neuropil.

The volume distribution of boutons synapsing on interneuron dendrites was significantly different than on relay dendrites (MW-U, *p* < 0.0001). Compared to those on relay cells, the size range of boutons on interneurons was narrower (0.01 to 21.1μm^3^ relay vs. 0.1 to 6.9μm^3^ interneuron), but the boutons were larger on average (mean volume: 1.1μm^3^ for relay vs. 1.7μm^3^ for interneuron), suggesting that interneurons receive more synapses from axons with large boutons (Figure 8H). Applying the cutoff criteria obtained from the unbiased dataset revealed that the smallest-sized DNM boutons were sparse (Figures 8F, G), contributing 4 and 5% of the inputs on interneurons. The retinal terminals contributed by far the largest number of synapses: close to 70% of all synapses on interneuron segments in our sample were formed by LM boutons (Figures 8E, G). This constitutes over 3 times and 10 times more retinal synapses on presumed X- and Y-type relay dendrites, respectively.

### 3.8. Selectivity of inputs on somata

While our sample did not include any soma that could be confirmed to give out dendrites with presynaptic F2 boutons, the relay cell dataset included several somatic segments that received synapses from reconstructed boutons (*n* = 72). The great majority of these boutons were of the three largest-sized DNM type (DM3-5; Figure 8I), confirming that inhibitory inputs dominate on the relay cell somata.

## 4. Discussion

The results of our study provided evidence that: (1) Relay cell and interneuron dendrites receive different relative amounts of retinal, inhibitory, cortical, and other modulatory synapses. While cortical and other modulatory synapses dominate relay cell inputs, interneuron inputs are primarily from retina. (2) Relay dendrites can be classified as belonging to X- and Y-type cell, based on the exclusive morphology for triads, and to some extent grape-like spine complexes, versus the dendrodendritic puncta adherentia. (3) X-type relay dendrites receive three times more retinal synapses than the Y-type dendrites. (4) Retinal and cortical synapses cluster and segregate on different segments of X-type dendrites. A similar selectivity is not evident for Y-type cells. (5) Volume-based classification of retinal boutons reveals at least four different bouton populations, suggesting different retinal ganglion cell types may have unique bouton morphology and size.

### 4.1. Methodological considerations

The methodology of the current study assumes that the sizes of axon boutons that originate from a cell population of a distinct phenotype are normally distributed. This assumption is intimated by many 2D TEM studies that revealed multiple peaks when all geniculate input boutons were plotted in frequency distribution histograms, suggesting that unique size distribution of individual inputs can be used for estimating area- or volume-based cutoff measures. However, the terminal area measurements obtained from 2D material can provide only an inference for the size of a roughly spherical bouton from any of its cross-sections, yielding a non-normal distribution even if the population is normally distributed. By utilizing 3D reconstructions and direct volume measurements of boutons in serial EM images, the current study aimed to identify potential volume-based cutoff criteria to sort geniculate inputs to distinct subpopulations of distinct origins.

To obtain an unbiased sample of geniculate boutons, we designed and utilized an approach for stereological sampling of synaptic boutons from SBEM image stacks. As is common in stereological approaches, our approach recognizes the heterogeneity of geniculate boutons (Sterio, 1984; Gundersen and Jensen, 1987; Mouton et al., 2002), and uses a random sampling design to take planar samples to account for a wide range of active zone sizes and the modularity of clustered boutons in the glomeruli. The cutoff values obtained from the unbiased dataset were suitable to mark the lowest overlap volumes between any two adjacent subpopulations both in the unbiased dataset and the datasets of terminal boutons synapsing on relay cell or interneuron dendrite segments, suggesting that the UTS can be a reliable approach for volume-sorting in a variety of tissues.

### 4.2. Morphological differences of X- and y-type relay cells

Relay cells in rodent LGN are morphologically distinct and have dendritic architectures that closely resemble X-, Y-, and W-cells of the cat. In rat LGN, geniculate relay cells are classified as bipolar, radial and basket cells, based on the dendrite branching patterns of cells retrogradely filled from the cortex (Ling et al., 2012). Similarly, in an extensive study employing a combined intracellular recording/filling approach (Krahe et al., 2011), mouse geniculate relay cells are described as displaying X-like (biconical), Y-like (symmetrical), or W-like (hemispheric) morphology. Furthermore, while all three cell types were mixed throughout the rat LGN, X-like and Y-like cells were found mixed in the ventral LGN (Krahe et al., 2011; Kerschensteiner and Guido, 2017; Guido, 2018), the same region from which the SBEM stacks used in the current study was obtained. Therefore, the current analysis assumed that morphologically distinct relay dendrites in the dataset belonged to X-like or Y-like (but not W-type) relay cells of the mouse LGN.

Past studies have also described fine morphological features that can distinguish X-like and Y-like relay dendrites. First, while thin, filopodia-like processes are encountered on all geniculate cell types, spines with large swellings and grape-like appendages are described as specific for most X-cells in cats, tree shrews, galago (Brauer et al., 1981; Friedlander et al., 1981; Conley et al., 1985; Humphrey and Weller, 1988) and for bipolar cells in the rats (Ling et al., 2012). In contrast, while simpler protrusions, such as filopodia or small spines, are observed occasionally on Y-cells, the grape-like appendages are rare (Friedlander et al., 1981; Wilson et al., 1984). Second, in an ultrastructural study of intracellularly filled X- and Y- cells in cat geniculate nucleus, Wilson and collegues demonstrated that triads involving retinal terminals, interneuron appendages and relay dendrites occurred frequently on X-cells, but rarely on Y-cell dendrites (Wilson et al., 1984). In categorizing the dendrite segments as X- or Y-like relay cells, we used the presence of triads vs. PA as the primary criteria because regardless that a dendrite emitted filopodia protrusions, spines or grape-like collection of spines, a dendrite arbor had either triads or PA, but not both.

Interestingly, all dendrite segments in the Y-like category displayed a specific morphological feature: dendrodendritic PA, which was not observed in X-like dendrites. Early ultrastructural studies of LGN describe puncta adherentia as a prominent formation at the large, primary input synapses in the thalamus (Colonnier and Guillery, 1964; Jones and Powell, 1969; Spacek and Lieberman, 1974); more recently, molecular properties of adherent junctions that occur at the excitatory synapses in the CA3 region of the hippocampus were studied in detail (Rollenhagen and Lübke, 2010; Wilke et al., 2013). When formed between an axon and a dendrite, the puncta adherentia or adherent junction structure is categorized as a filamentous contact, characterized by co-extensive stretches of apposed axonal and dendritic plasma membranes; electrondense paramembrenous material that forms a reticulum on each side of the cleft-like intercellular space, but more densely on the dendrite side of the apposition (Colonnier and Guillery, 1964; Spacek and Lieberman, 1974; Bickford et al., 2010; Hammer et al., 2014). The presence of such filamentous axodendritic contacts is found not correlated with morphological cell types (biconical vs. saucer-shaped) in the mouse LGN (Morgan et al., 2016). A prominent characteristic of the dendritic side of the axo-dendritic PA is a reticulum of fine filaments and an elaborate system of tubules of smooth ER (Colonnier and Guillery, 1964), suggesting that filamentous contacts may trigger local ionic or molecular events that involve the ER. Regardless, axo-dendritic PA or filamentous contacts are regarded as contributing to the stability of the synapse due to cell adhesion molecules that may be present there (also see below).

The puncta adherentia structure was also described as occurring between dendrites: While extensively described by Peter and Palay (Peters and Palay, 1966) in their original characterization of geniculate ultrastructural morphology, the dendrodendritic PA has not been mentioned as prominently in more recent literature, perhaps due to unfavorable fixation or counterstain conditions commonly used for TEM embedding. Alternatively, 2D analyses may bias the experimenters’ attention to synapses rather than synapse-barren regions of the dendrite shafts. The SBEM preparation and our reconstruction strategy centered on dendrite segments, have been favorable to revealing frequent occurrence of dendrodendritic and somatosomatic PAs involving geniculate relay cells. Furthermore, we have revealed that dendrodendritic PAs occur exclusively between dendrites that are devoid of grape-like spine arrangements, glomeruli and triads, the identifying characteristics of X-type relay cells. As such, we propose that dendrodendritic PAs are unique markers for Y-type relay cells in the mouse LGN.

The potential function of PA has been studied in the thalamus and the hippocampus. At the CA3 synapse, PA junctions are associated with cell adhesion molecule nectin, thus proposed to serve an adhesion function between the mossy terminals and the postsynaptic dendrites (Rollenhagen et al., 2007; Maruo et al., 2018). A similar function is conceivable for PAs of the retinogeniculate synapses. On the other hand, the presence of smooth ER at both sides of the dendrodendritic PA may also indicate a potential function for the PA beyond adhering two dendrites together. A possibility is that paramembrenous ER may provide a site for Ca^+2^ signaling, suggesting a mechanism for non-chemical synapse regulated activity coupling among Y-type relay cells. A form of high-frequency burst firing coupling is encountered in a subpopulation of geniculate relay cells. This type of high-frequency burst coupling is impervious to TTX but abolished by gap junction blockers; the cells that show high-frequency burst coupling also display dye coupling (Hughes et al., 2004) between cells that have overlapping dendritic arbor spread (see Figure 7G in Hughes et al., 2004). While detection of the classical gap junction morphology has not been possible at SBEM image resolution, and successful immuno-EM labeling with Cx36 antibodies has been elusive due to deterioration of labeling even with the light fixation procedures needed to preserve ultrastructure (Rubio and Nagy, 2015), direct evidence for dendrodendritic gap junctions is present in the cat brain (Hughes et al., 2011) and moderate levels of neuronal connexon expression is found in mouse geniculate cells (Allen Mouse Brain Atlas, https://mouse.brain-map.org/experiment/show/71836902). The possibility that dendrodendritic PAs can be a site of connexin hemichannels is intriguing.

### 4.3. Synaptic circuitry on relay cells vs. interneurons

Earlier 2D TEM studies have suggested that corticothalamic axons have terminals that are the smallest in size, while cholinergic inputs are only slightly larger, and these two terminal populations make up about 60% of all LGN terminals (Montero, 1991; Erişir et al., 1997a,b; Sherman and Guillery, 2001). The GABAergic terminals, including from GABAergic interneurons and neurons from the thalamic reticular nucleus (TRN), are medium in size, making up about 25–35% of LGN inputs (Montero, 1991; Erişir et al., 1998). Finally, earlier 2D TEM studies report that the axons originating from the retina have the largest terminal boutons found in the LGN, and they provide as much as 20%, and as little as 5% of synapses in the geniculate circuitry (Guillery, 1969b; Wilson et al., 1984; Montero, 1991; Van Horn et al., 2000; Sherman and Guillery, 2001). The 3D volume sorting approach utilized in the current study matched and confirmed these findings and provided further granularity on how these input distributions differ for distinct geniculate cell-types. For example, the current study reveals that three distinct geniculate cell types (putative X-, Y- and interneuron) receive 27, 7 and 68% of their synaptic inputs from the retina, respectively. Similarly, the smallest-sized boutons, including corticothalamic and cholinergic inputs, which provide the largest subpopulation of synapses on relay cells, constitute a small portion of inputs on the interneuron dendrite segments. To note, this finding conflicts with what was described for cat A-laminae (Erişir et al., 1998), where small cross-section terminals (classified as RD-type) constitute about 1/3 of inputs onto interneurons, in contrast to about 10% found in our sample. This discrepancy can be explained by how the interneurons were identified in each study, by potential species differences, by the existence of multiple interneuron types, or by the combination of all these factors. First, based on morphological, histochemical, and physiological findings, the existence of at least two types of interneurons has been demonstrated in mice (Leist et al., 2016), rats (Gabbott and Bacon, 1994), cats (Bickford et al., 1999) and ferrets (Sanchez-Vives et al., 1996). If these interneuron types are represented in the current SBEM samples, and each receives a different composition of inputs, our data from a limited number of dendrite reconstructions may not have resolved this granularity. Second, the current study of mouse LGN identified the interneuron dendrites based on whether they form presynaptic zones. In contrast, the cat study utilized GABA immunolabeling, which may have facilitated the inclusion of interneuron dendrite branches that may be devoid of presynaptic zones as well. While GABA immunolabeling in 3D image stack preparations in both species can resolve this discrepancy, it is currently not feasible to conclude whether the methodological or species differences can be the culprit. Finally, a recent 3D reconstruction of a mouse interneuron in its entirety provided evidence that over 2/3 of inputs come from retinal axons, which were identified by their RLP morphology (Morgan and Lichtman, 2020). Similar to ours, this study has used the presence of F2 morphology as the identifying criterion for classifying the cell as an interneuron. The reconstructed interneuron was also located in a comparable LGN region as our SBEM samples. The similarities in the innervation patterns between the Morgan and Lichtman interneuron and our sample of interneuron dendrite segments from the same region strengthen our conclusions that mice interneurons receive robust excitation from the retina and considerably sparse modulatory inputs from the cortical and the brainstem.

The current study also reveals that at least four distinct retinal inputs contribute to the geniculate circuitry, providing evidence for morphological signatures that may mark the different RGC-types projecting to the LGN (Sanes and Masland, 2015; Baden et al., 2016; Martersteck et al., 2017). While the current study does not render any data to support the idea that individual retinal axons yield only boutons that fit the size distribution of one LM-subpopulation because only the boutons, but not their axons, were reconstructed, our findings is consistent with the extensive reconstruction study of Morgan and colleagues (Morgan et al., 2016), which revealed that individual retinal axons bore terminal boutons with similar (i.e., small or large) cross-section sizes. The statistical sorting of bouton volumes developed in the current study may now provide an opportunity for re-examining bouton volumes in wider datasets reconstructing individual retinal axons.

### 4.4. Synaptic selectivity on X-type and y-type relay cells and interneurons

The results demonstrate that axons originating from different brain regions have selectivity for distinct dendrite branches: while retinal boutons are exclusively compartmentalized on the primary and secondary dendrite branches, corticothalamic boutons only target tertiary branches or the dendrite segment that are distal to the last branching point of a cell. Furthermore, this target selectivity feature is a property of X-type relay cells but not of Y-type relay cells or interneurons. That the retinal and corticothalamic synapses are preferentially located on proximal and distal relay dendrites, respectively, was indeed suggested in earlier TEM studies (Wilson et al., 1984; Hamos et al., 1987; Erişir et al., 1997a; Bickford, 2015). In particular, Hamos and colleagues (Hamos et al., 1987), who reconstructed an intracellularly filled X-type retinal axon and the relay cells it contacted across 1,200 serial sections at the TEM resolution several decades before the current 3D reconstruction techniques were developed, noted that retinal terminals synapsed mostly on appendages and proximal dendrite shafts of three out of four postsynaptic relay cells they examined. The fourth cell, the soma size of which lay within the range of Y-cells, received retinal synapses on distal dendrites as well. Our results now confirm that retinal synapses cluster on primary and secondary dendrites, regardless of the caliber of the dendrite, and that this is an exclusive property of relay cells with X-type morphology. The current study also provides evidence that corticothalamic synapses are fully compartmentalized on tertiary branches of X-type relay cells, which are devoid of retinal inputs.

## Data availability statement

The datasets presented in this study can be found in the online repository, BossDB.org with the following accession link: https://bossdb.org/project/maher_briegel2023.

## Ethics statement

This animal study was reviewed and approved by IACUC Virginia Tech.

## Author contributions

EM and AB supervised and contributed to the reconstructions, analyzed data, ran statistical models, contributed to the first draft of the manuscript, and revised the figures and the manuscript. SI contributed to the reconstructions. MF provided the SBEM stacks used in the study and contributed to the writing. HG designed and supervised the analyses with statistical modeling and contributed to the writing. AE designed the experiments, supervised and performed the analyses, prepared figures, and wrote the manuscript. All authors contributed to the article and approved the submitted version.

## Supplementary material

Supplementary Figure 1 Geniculate relay dendrites examples of geniculate relay cell dendrite segments reconstructed from two SBEM image stacks. The segments were identified as primary if they emerged from a cell body **(a–f)**, or displayed fragments of endoplasmic reticulum or Golgi apparatus. Fine processes and appendages are commonly found. The scale-cubes represent 1 m × 1 m.

Supplementary Figure 2 A series of SBEM images displaying a relay dendrite segment that gives off a grape-like appendage. The series represents a 2.5 micron thickness of the tissue. Every second image in the sequence, numbered 74 to 108, is organized in successive columns. The cross-sections of a relay cell dendrite are traced and pseudocolored blue. This dendrite gives off a thin stalk (around section 90), subsequently giving off three large swellings (or grapes). Each grape receives synapses from multiple boutons. For example, the grape in section 90 receives three synapses (red lines): one from a retinal terminal (pseudocolored light green), a second one from a large, dark mitochondria-containing, presumed inhibitory cell terminal (red), and a third one from a small-sized bouton that does not contain mitochondria. The latter bouton was classified as presumed brainstem origin. The second grape, visible in sections 74 to 88, receives synapses from 4 boutons: two retinal terminals (light and dark green), and two medium-sized, presumed inhibitory terminals (orange and red). A third grape in sections 96 to 108 receives synapses from 3 retinal terminals (all light green) and one inhibitory bouton (red). Notice that this red bouton also receives synapses from two of these retinal boutons (in sections 102 and 104), thereby forming a complex triad involving the grape. Because the red bouton is both presynaptic and postsynaptic, it is classified as an F2 bouton, the dendritic appendage of a geniculate interneuron.

Supplementary Figure 3 Geniculate input boutons display a large range of sizes. Reconstructions **(A,C,E,G,I,K,M)** and a representative image of the section for each reconstruction (**B,D,F,H,J,L,N**, respectively) are paired in adjacent columns. The boutons that originate from retinal ganglion cells are identified by their unique mitochondrial morphology (panels **K–N**): the mitochondria appear pale or with light contrast, primarily owing to larger cristae and the cytoplasm that is lightly electron dense. While a wide range of bouton sizes are found, retinal boutons provide the largest-sized boutons in the geniculate circuitry. The boutons that contain no (panels **A,B**) or dark mitochondria (panels **C–J**) also represent a very wide range and include corticothalamic, brainstem, interneuron, and thalamic reticular nucleus inputs. Notice that the largest-sized non-retinal boutons can be within the size range of the largest retinal terminals. Scale bars in **(B,N)** = 500 nm, and apply to all panels.

## References

[B1] Baden T. Berens P. Franke K. Román Rosón M. Bethge M. Euler T. (2016). The functional diversity of retinal ganglion cells in the mouse. *Nature* 529 345–350. 10.1038/nature16468 26735013 PMC4724341

[B2] Balaram P. Isaamullah M. Petry H. M. Bickford M. E. Kaas J. H. (2015). Distributions of vesicular glutamate transporters 1 and 2 in the visual system of tree shrews (*Tupaia belangeri*). *J. Comp. Neurol.* 523 1792–1808. 10.1002/cne.23727 25521420 PMC4470886

[B3] Bickford M. E. (2015). Thalamic circuit diversity: Modulation of the driver/modulator framework. *Front. Neural Circuits* 9:86. 10.3389/fncir.2015.00086 26793068 PMC4709853

[B4] Bickford M. E. (2019). Synaptic organization of the dorsal lateral geniculate nucleus. *Eur. J. Neurosci.* 49 938–947. 10.1111/EJN.13917 29575193 PMC6157005

[B5] Bickford M. E. Ramcharan E. Godwin D. W. Erisir A. Gnadt J. Sherman S. M. (2000). Neurotransmitters contained in the subcortical extraretinal inputs to the monkey lateral geniculate nucleus. *J. Comp. Neurol.* 424 701–717. 10.1002/1096-9861(20000904)424:4<701::AID-CNE11<3.0.CO;2-B 10931491

[B6] Bickford M. E. Slusarczyk A. Dilger E. K. Krahe T. E. Kucuk C. Guido W. (2010). Synaptic development of the mouse dorsal lateral geniculate nucleus. *J. Comp. Neurol.* 518 622–635. 10.1002/cne.22223 20034053 PMC4278806

[B7] Bickford M. E. Wei H. Eisenback M. A. Chomsung R. D. Slusarczyk A. S. Dankowsi A. B. (2008). Synaptic organization of thalamocortical axon collaterals in the perigeniculate nucleus and dorsal lateral geniculate nucleus. *J. Comp. Neurol* 508 264–285. 10.1002/cne.21671 18314907 PMC2561320

[B8] Bickford M. Carden W. Patel N. (1999). Two types of interneurons in the cat visual thalamus are distinguished by morphology, synaptic connections, and nitric oxide synthase content. *J. Comp. Neurol.* 413 83–100. 10464372

[B9] Boey A. Rybakin V. Kalicharan D. Vints K. Gounko N. (2019). Gold-substituted Silver-intensified peroxidase immunolabeling for FIB-SEM Imaging. *J. Histochem. Cytochem.* 67 351–360. 10.1369/0022155418824335 30624131 PMC6495490

[B10] Brauer K. Werner L. Winkelmann E. Luth H. J. (1981). The dorsal lateral geniculate nucleus of *Tupaia glis*: A Golgi, Nissl and acetylcholinesterase study. *J. Hirnforsch.* 22 59–74. 7240727

[B11] Campbell X. P. W. Govindaiah G. Masterson S. P. Bickford X. M. E. Guido W. (2020). Synaptic properties of the feedback connections from the thalamic reticular nucleus to the dorsal lateral geniculate nucleus. *J. Neurophysiol.* 124 404–417. 10.1152/jn.00757.2019 32609582 PMC7500366

[B12] Çavdar S. Hacioǧlu H. Şirvanci S. Keskinöz E. Onat F. (2011). Synaptic organization of the rat thalamus: A quantitative study. *Neurol. Sci.* 32 1047–1056. 10.1007/s10072-011-0606-4 21544663

[B13] Colonnier M. Guillery R. W. (1964). Synaptic organization in the lateral geniculate nucleus of the monkey. *Z. Zellforsch. Mikrosk. Anat.* 62 333–355. 10.1007/BF00339284 14218147

[B14] Conley M. Birecree E. Casagrande V. A. (1985). Neuronal classes and their relation to functional and laminar organization of the lateral geniculate nucleus: A Golgi study of the prosimian primate, Galago crassicaudatus. *J. Comp. Neurol.* 242 561–583. 10.1002/cne.902420407 2418081

[B15] Crandall S. R. Cox C. L. (2013). Thalamic microcircuits: Presynaptic dendrites form two feedforward inhibitory pathways in thalamus. *J. Neurophysiol.* 110 470–480. 10.1152/jn.00559.2012 23615551 PMC3727071

[B16] Dankowski A. Bickford M. E. (2003). Inhibitory circuitry involving Y cells and Y retinal terminals in the C laminae of the cat dorsal lateral geniculate nucleus. *J. Comp. Neurol.* 460 368–379. 10.1002/cne.10640 12692855

[B17] Datskovskaia A. Breckinridge C. Bickford M. E. (2001). Y retinal terminals contact interneurons in the cat dorsal lateral geniculate nucleus. *J. Comp. Neurol.* 100 85–100. 10.1002/1096-9861(20010129)430:1<85::aid-cne1016>3.0.co;2-k 11135247

[B18] Denk W. Horstmann H. (2004). Serial block-face scanning electron microscopy to reconstruct three-dimensional tissue nanostructure. *PLoS Biol.* 2:e329. 10.1371/journal.pbio.0020329 15514700 PMC524270

[B19] Dreher B. Sefton A. J. Ni S. Y. K. Nisbelt G. (1985). The morphology, number, distribution and central projections of Class I retinal ganglion cells in albino and hooded rats. *Brain Behav. Evol.* 26 10–48. 10.1159/000118764 3902145

[B20] Epskamp S. Cramer A. O. J. Waldorp L. J. Schmittmann V. D. Borsboom D. (2012). qgraph: Network visualizations of relationships in psychometric data. *J. Stat. Softw.* 48 1–18. 10.18637/JSS.V048.I04

[B21] Erişir A. Van Horn S. C. Bickford M. E. Sherman S. M. (1997a). Immunocytochemistry and distribution of parabrachial terminals in the lateral geniculate nucleus of the cat: A comparison with corticogeniculate terminals. *J. Comp. Neurol.* 377 535–549. 9007191

[B22] Erişir A. Van Horn S. C. Sherman S. M. (1997b). Relative numbers of cortical and brainstem inputs to the lateral geniculate nucleus. *Proc. Natl. Acad. Sci. U.S.A.* 94 1517–1520. 10.1073/pnas.94.4.1517 9037085 PMC19823

[B23] Erişir A. Van Horn S. C. Sherman S. M. (1998). Distribution of synapses in the lateral geniculate nucleus of the cat: Differences between laminae A and A1 and between relay cells and interneurons. *J. Comp. Neurol.* 390 247–255. 9453668

[B24] Fiala J. C. (2005). Reconstruct: A free editor for serial section microscopy. *J. Microsc.* 218 52–61. 10.1111/j.1365-2818.2005.01466.x 15817063

[B25] Fitzpatrick D. Diamond I. Raczkowski D. (1989). Cholinergic and monoaminergic innervation of the cat’s thalamus: comparison of the lateral geniculate nucleus with other principal sensory nuclei. *J. Comp. Neurol.* 288 647–675. 10.1002/cne.902880411 2478594

[B26] Friedlander M. J. Lin C. S. Stanford L. R. Sherman S. M. (1981). Morphology of functionally identified neurons in lateral geniculate nucleus of the cat. *J. Neurophysiol.* 46 80–129. 10.1152/jn.1981.46.1.80 7264710

[B27] Gabbott P. Bacon S. (1994). Two types of interneuron in the dorsal lateral geniculate nucleus of the rat: a combined NADPH diaphorase histochemical and GABA immunocytochemical study. *J. Comp. Neurol.* 350 281–301. 10.1002/cne.903500211 7884043

[B28] Guido W. (2018). Development, form, and function of the mouse visual thalamus. *J. Neurophysiol.* 120 211–225. 10.1152/jn.00651.2017 29641300 PMC6093956

[B29] Guillery R. W. (1969a). The organization of synaptic interconnections in the laminae of the dorsal lateral geniculate nucleus of the cat. *Z. Zellforsch. Mikrosk. Anat.* 96 1–38. 10.1007/BF00321474 5772028

[B30] Guillery R. W. (1969b). A quantitative study of synaptic interconnections in the dorsal lateral geniculate nucleus of the cat. *Z. Zellforsch. Mikrosk. Anat.* 96 39–48. 10.1007/BF00321474 5772028

[B31] Guillery R. W. (1970). The laminar distribution of retinal fibers in the dorsal lateral geniculate nucleus of the cat: A new interpretation. *J. Comp. Neurol.* 138 339–367. 10.1002/cne.901380307 5442835

[B32] Gundersen H. J. G. Jensen E. B. (1987). The efficiency of systematic sampling in stereology and its prediction. *J. Microsc.* 147 229–263. 10.1111/j.1365-2818.1987.tb02837.x 3430576

[B33] Hahsler M. Hornik K. (2009). arules – A computational environment for mining association rules and frequent item sets. *J. Stat. Softw.* 14 1–25.

[B34] Hahsler M. Grun B. Hornik K. (2005). arules – A computational environment for mining association rules and frequent item sets. *J. Stat. Softw.* 14 1–6.

[B35] Hammer S. Carrillo G. L. Govindaiah G. Monavarfeshani A. Bircher J. S. Su J. (2014). Nuclei-specific differences in nerve terminal distribution, morphology, and development in mouse visual thalamus. *Neural Dev.* 9 1–20. 10.1186/1749-8104-9-16 25011644 PMC4108237

[B36] Hammer S. Monavarfeshani A. Lemon T. Su J. Fox M. A. Andrew M. (2015). Multiple retinal axons converge onto relay cells in the adult mouse thalamus. *Cell Rep.* 12 1575–1583. 10.1016/j.celrep.2015.08.003 26321636 PMC5757867

[B37] Hamos J. E. Van Horn S. C. Raczkowski D. Sherman S. M. (1987). Synaptic circuits involving an individual retinogeniculate axon in the cat. *J. Comp. Neurol.* 259 165–192. 10.1002/cne.902590202 3584556

[B38] Hamos J. Van Horn S. Raczkowski D. Uhlrich D. Sherman S. (1985). Synaptic connectivity of a local circuit neurone in lateral geniculate nucleus of the cat. *Nature.* 317 618–621. 10.1038/317618a0 4058571

[B39] Harary F. (1969). *Graph theory.* New York, NY: Addison-Wesley Publishing Company.

[B40] Helmstaedter M. Briggman K. L. Denk W. (2008). 3D structural imaging of the brain with photons and electrons. *Curr. Opin. Neurobiol.* 18 633–641. 10.1016/j.conb.2009.03.005 19361979

[B41] Hughes S. W. Lorincz M. L. Blethyn K. Kékesi K. A. Juhász G. Turmaine M. (2011). Thalamic gap junctions control local neuronal synchrony and influence macroscopic oscillation amplitude during EEG alpha rhythms. *Front. Psychol.* 2:193. 10.3389/FPSYG.2011.00193/BIBTEX PMC3187667 22007176

[B42] Hughes S. W. Lörincz M. Cope D. W. Blethyn K. L. Kékesi K. A. Parri H. R. (2004). Synchronized oscillations at α and θ frequencies in the lateral geniculate nucleus. *Neuron* 42 253–268. 10.1016/S0896-6273(04)00191-6 15091341

[B43] Humphrey A. L. Weller R. E. (1988). Structural correlates of functionally distinct X-cells in the lateral geniculate nucleus of the cat. *J. Comp. Neurol.* 268 448–468. 10.1002/cne.902680312 3360998

[B44] Jones E. G. (2002). Thalamic organization and function after Cajal. *Prog. Brain Res.* 136 333–357. 10.1016/S0079-6123(02)36029-1 12143393

[B45] Jones E. G. Powell T. P. (1969). Electron microscopy of synaptic glomeruli in the thalamic relay nuclei of the cat. *Proc. R. Soc. London. Ser. B. Biol. Sci.* 172 153–171. 10.1098/rspb.1969.0017 4388107

[B46] Kerschensteiner D. Guido W. (2017). Organization of the dorsal lateral geniculate nucleus in the mouse. *Vis. Neurosci.* 34:E008. 10.1017/S0952523817000062 28965501 PMC6380502

[B47] Krahe T. E. El-Danaf R. N. Dilger E. K. Henderson S. C. Guido W. (2011). Morphologically distinct classes of relay cells exhibit regional preferences in the dorsal lateral geniculate nucleus of the mouse. *J. Neurosci.* 31 17437–17448. 10.1523/JNEUROSCI.4370-11.2011 22131405 PMC6623799

[B48] Leist M. Datunashvilli M. Kanyshkova T. Zobeiri M. Aissaoui A. Cerina M. (2016). Two types of interneurons in the mouse lateral geniculate nucleus are characterized by different h-current density. *Sci Rep.* 6:24904. 10.1038/srep24904 27121468 PMC4848471

[B49] Ling C. Hendrickson M. L. Kalil R. E. (2012). Morphology, classification, and distribution of the projection neurons in the dorsal lateral geniculate nucleus of the rat. *PLoS One* 7:e49161. 10.1371/journal.pone.0049161 23139837 PMC3489731

[B50] Luján R. Merchán-Pérez A. Soriano J. Martín-Belmonte A. Aguado C. Alfaro-Ruiz R. (2021). Neuron class and target variability in the three-dimensional localization of SK2 channels in hippocampal neurons as detected by immunogold FIB-SEM. *Front Neuroanat.* 15:781314. 10.3389/fnana.2021.781314 34975419 PMC8715088

[B51] Mantyh P. W. Kemp J. A. (1983). The distribution of putative neurotransmitters in the lateral geniculate nucleus of the rat. *Brain Res.* 288 344–348. 10.1016/0006-8993(83)90115-4 6198031

[B52] Martersteck E. M. Hirokawa K. E. Evarts M. Bernard A. Duan X. Li Y. (2017). Diverse central projection patterns of retinal ganglion Cells. *Cell Rep.* 18 2058–2072. 10.1016/j.celrep.2017.01.075 28228269 PMC5357325

[B53] Maruo T. Sakakibara S. Miyata M. Itoh Y. Kurita S. Mandai K. (2018). Involvement of l-afadin, but not s-afadin, in the formation of puncta adherentia junctions of hippocampal synapses. *Mol. Cell. Neurosci.* 92 40–49. 10.1016/j.mcn.2018.06.006 29969655

[B54] McCormick D. A. Prince D. A. (1987). Actions of acetylcholine in the guinea-pig and cat medial and lateral geniculate nuclei, in vitro. *J. Physiol.* 392 147–165. 10.1113/jphysiol.1987.sp016774 2833597 PMC1192298

[B55] Montero V. (1986). Localization of gamma-aminobutyric acid (GABA) in type 3 cells and demonstration of their source to F2 terminals in the cat lateral geniculate nucleus: a Golgi-electron-microscopic GABA-immunocytochemical study. *J Comp Neurol.* 254 228–245. 10.1002/cne.902540207 3540041

[B56] Montero V. M. (1991). A quantitative study of synaptic contacts on interneurons and relay cells of the cat lateral geniculate nucleus. *Exp. Brain Res.* 86 257–270. 10.1007/BF00228950 1756802

[B57] Morgan J. L. Lichtman J. W. (2020). An individual interneuron participates in many kinds of inhibition and innervates much of the mouse visual thalamus. *Neuron* 106 468–481.e2. 10.1016/J.NEURON.2020.02.001 32142646 PMC7295017

[B58] Morgan J. L. Berger D. R. Wetzel A. W. Lichtman J. W. (2016). The fuzzy logic of network connectivity in mouse visual thalamus. *Cell* 165 192–206. 10.1016/j.cell.2016.02.033 27015312 PMC4808248

[B59] Mouton P. R. Gokhale A. M. Ward N. L. West M. J. (2002). Stereological length estimation using spherical probes. *J. Microsc.* 206 54–64. 12000563 10.1046/j.1365-2818.2002.01006.x

[B60] Novotny G. (1979). Observations on the lateral geniculate nucleus of the monkey (*Macaca fascicularis*) after eye removal: a light and electron microscopic study. I. Classification and degeneration of optic fibre terminals. *J Hirnforsch* 20 561–580. 121133

[B61] Ohara P. T. Chazal G. Ralston H. J. (1989). Ultrastructural analysis of gaba-immunoreactive elements in the monkey thalamic ventrobasal complex. *J. Comp. Neurol.* 283 541–558. 10.1002/cne.902830408 2745753

[B62] Peters A. Palay S. L. (1966). The morphology of laminae A and Al of the dorsal nucleus of the lateral geniculate body of the cat. *J. Anat* 103 451–486. PMC1270792 5965439

[B63] Rapisardi S. Miles T. (1984). Synaptology of retinal terminals in the dorsal lateral geniculate nucleus of the cat. *J Comp Neurol.* 223 515–534. 10.1002/cne.902230405 6715570

[B64] Rollenhagen A. Lübke J. H. R. (2010). The mossy fi ber bouton: The “ common ” or the “ unique ” synapse? *Front Synaptic Neurosci.* 2:2. 10.3389/fnsyn.2010.00002 21423488 PMC3059708

[B65] Rollenhagen A. Sätzler K. Rodríguez E. P. Jonas P. Frotscher M. Lübke J. H. R. (2007). Structural determinants of transmission at large hippocampal mossy fiber synapses. *J. Neurosci.* 27 10434–10444. 10.1523/JNEUROSCI.1946-07.2007 17898215 PMC6673150

[B66] Rovó Z. Ulbert I. Acsády L. (2012). Drivers of the primate thalamus. *J. Neurosci.* 32 17894–17908. 10.1523/JNEUROSCI.2815-12.2012 23223308 PMC3672843

[B67] Rubio M. E. Nagy J. I. (2015). Connexin36 expression in major centers of the auditory system in the CNS of mouse and rat: Evidence for neurons forming purely electrical synapses and morphologically mixed synapses. *Neuroscience* 303:604. 10.1016/J.NEUROSCIENCE.2015.07.026 26188286 PMC4576740

[B68] Sanchez-Vives M. Bal T. Kim U. von Krosigk M. McCormick D. (1996). Are the interlaminar zones of the ferret dorsal lateral geniculate nucleus actually part of the perigeniculate nucleus? *J. Neuroscience.* 16 5923–5941. 10.1523/JNEUROSCI.16-19-05923.1996 8815875 PMC6579195

[B69] Sanes J. R. Masland R. H. (2015). The types of retinal ganglion cells: Current status and implications for neuronal classification. *Annu. Rev. Neurosci.* 38 221–246. 10.1146/annurev-neuro-071714-034120 25897874

[B70] Scrucca L. Fop M. Murphy T. B. Raftery A. (2016). mclust 5: Clustering, classification and density estimation using gaussian finite mixture models. *R J.* 8:289. 10.32614/RJ-2016-021 PMC5096736 27818791

[B71] Sherman S. M. (2004). Interneurons and triadic circuitry of the thalamus. *Trends Neurosci.* 27 670–675. 10.1016/j.tins.2004.08.003 15474167

[B72] Sherman S. M. Guillery R. W. (1996). Functional organization of thalamocortical relays. *J. Neurophysiol.* 76 1367–1395. 8890259 10.1152/jn.1996.76.3.1367

[B73] Sherman S. M. Guillery R. W. (1998). On the actions that one nerve cell can have on another: Distinguishing “drivers” from “modulators”. *Proc. Natl. Acad. Sci. U.S.A.* 95 7121–7126. 10.1073/pnas.95.12.7121 9618549 PMC22761

[B74] Sherman S. M. Guillery R. W. (2001). *Exploring the thalamus.* San Diego, CA: Academic Press.

[B75] Spacek J. Lieberman A. (1974). Ultrastructure and three-dimensional organization of synaptic glomeruli in rat somatosensory thalamus. *J. Anat.* 117 487–516. 4370696 PMC1231457

[B76] Sterio D. C. (1984). The unbiased estimation of number and sizes of arbitrary particles using the disector. *J. Microsc.* 134 127–136. 10.1111/j.1365-2818.1984.tb02501.x 6737468

[B77] Szentágothai J. Hámori J. Tömböl T. (1966). Degeneration and electron microscope analysis of the synaptic glomeruli in the lateral geniculate body. *Exp Brain Res.* 2 283–301. 10.1007/BF00234775 5957903

[B78] Van Horn S. C. Erisir A. Sherman S. M. (2000). Relative distribution of synapses in the A-laminae of the lateral geniculate nucleus of the cat. *J. Comp. Neurol.* 416 509–520. 10.1002/(SICI)1096-9861(20000124)416:4<509::AID-CNE7<3.3.CO;2-8 10660881

[B79] Wang S. Bickford M. E. Van Horn S. C. Erisir A. Godwin D. W. Sherman S. M. (2001). Synaptic targets of thalamic reticular nucleus terminals in the visual thalamus of the cat. *J. Comp. Neurol.* 440 321–341. 10.1002/cne.1389 11745627

[B80] Wang S. Eisenback M. Datskovskaia A. Boyce M. Bickford M. (2002). GABAergic pretectal terminals contact GABAergic interneurons in the cat dorsal lateral geniculate nucleus. *Neurosci. Lett.* 323 141–145. 10.1016/s0304-3940(01)02533-2 11950513

[B81] Wilke S. A. Antonios J. K. Bushong E. A. Badkoobehi A. Malek E. Hwang M. (2013). Deconstructing complexity: Serial block-face electron microscopic analysis of the hippocampal mossy fiber synapse. *J. Neurosci.* 33 507–522. 10.1523/JNEUROSCI.1600-12.2013 23303931 PMC3756657

[B82] Wilson J. R. (1989). Synaptic organization of individual neurons in the macaque lateral geniculate nucleus. *J. Neurosci.* 9 2931–2953. 10.1523/JNEUROSCI.09-08-02931 2769372 PMC6569706

[B83] Wilson J. R. Friedlander M. J. Sherman S. M. (1984). Fine structural morphology of identified X- and Y-cells in the cat’s lateral geniculate nucleus. *Proc. R. Soc. Lond. Ser. B Biol. Sci.* 221 411–436. 10.1098/rspb.1984.0042 6146984

