# Supplementary figures and images for: 3D electron microscopy and volume-based bouton sorting reveal the selectivity of inputs onto geniculate relay cell and interneuron dendrite segments
Source: Front Neuroanat. 2023 Mar 17;17:1150747. doi: 10.3389/fnana.2023.1150747 (PMC10064015; doi:10.3389/fnana.2023.1150747)

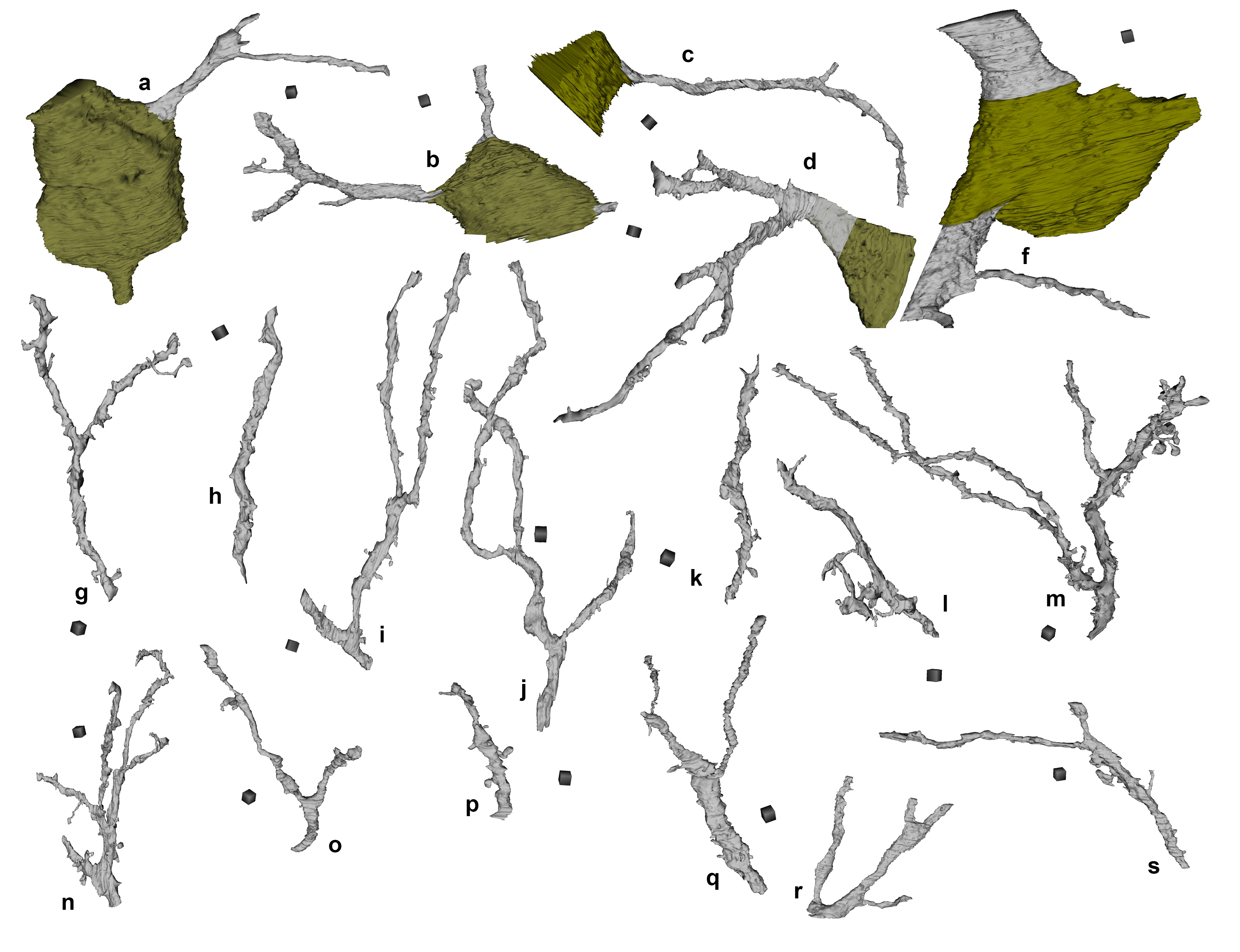

Supplement: Supplementary Figure 1 — Geniculate relay dendrites examples of geniculate relay cell dendrite segments reconstructed from two SBEM image stacks. The segments were identified as primary if they emerged from a cell body (a–f), or displayed fragments of endoplasmic reticulum or Golgi apparatus. Fine processes and appendages are commonly found. The scale-cubes represent 1 m × 1 m. [file Image_1.jpg]

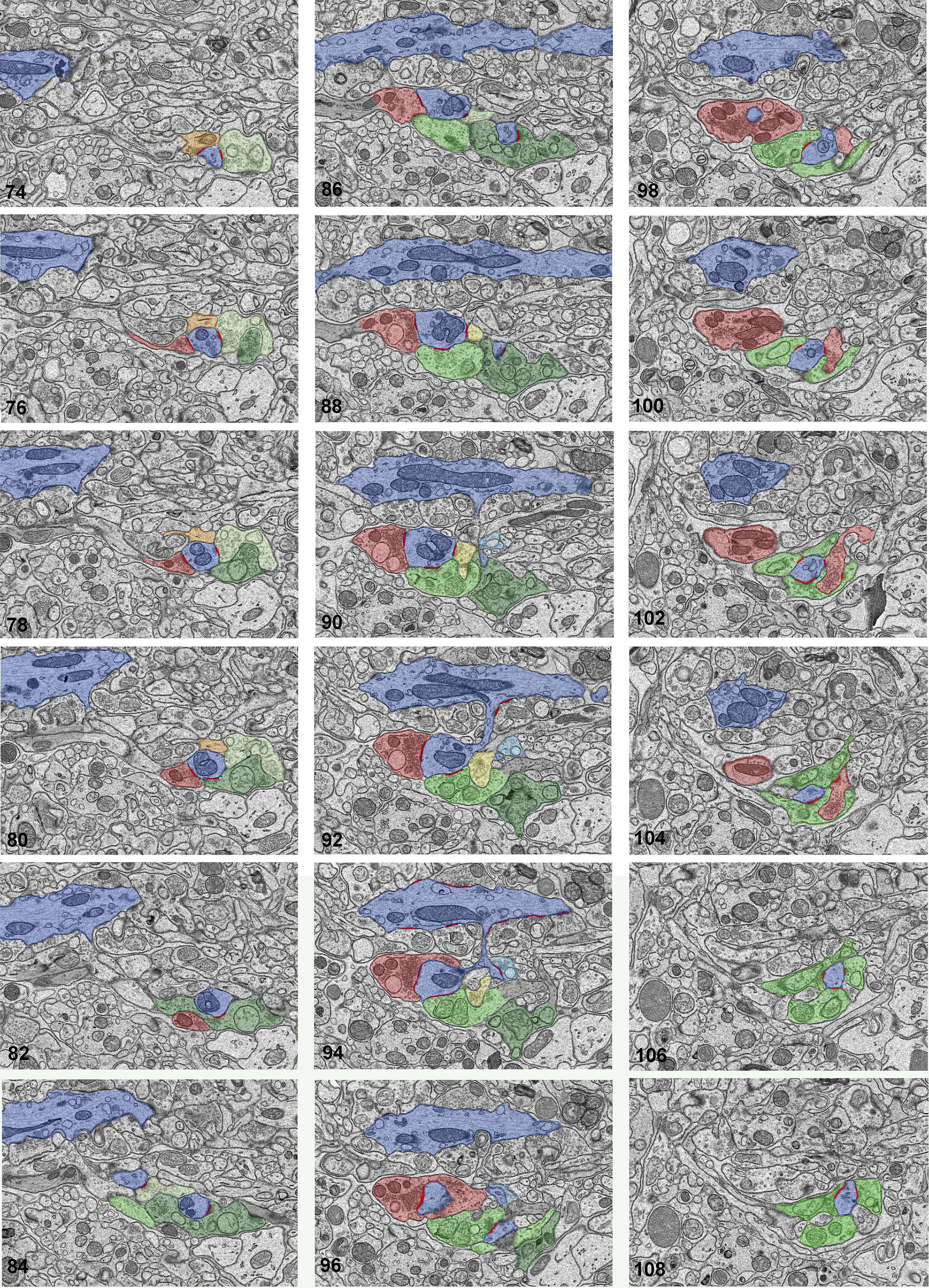

Supplement: Supplementary Figure 2 — A series of SBEM images displaying a relay dendrite segment that gives off a grape-like appendage. The series represents a 2.5 micron thickness of the tissue. Every second image in the sequence, numbered 74 to 108, is organized in successive columns. The cross-sections of a relay cell dendrite are traced and pseudocolored blue. This dendrite gives off a thin stalk (around section 90), subsequently giving off three large swellings (or grapes). Each grape receives synapses from multiple boutons. For example, the grape in section 90 receives three synapses (red lines): one from a retinal terminal (pseudocolored light green), a second one from a large, dark mitochondria-containing, presumed inhibitory cell terminal (red), and a third one from a small-sized bouton that does not contain mitochondria. The latter bouton was classified as presumed brainstem origin. The second grape, visible in sections 74 to 88, receives synapses from 4 boutons: two retinal terminals (light and dark green), and two medium-sized, presumed inhibitory terminals (orange and red). A third grape in sections 96 to 108 receives synapses from 3 retinal terminals (all light green) and one inhibitory bouton (red). Notice that this red bouton also receives synapses from two of these retinal boutons (in sections 102 and 104), thereby forming a complex triad involving the grape. Because the red bouton is both presynaptic and postsynaptic, it is classified as an F2 bouton, the dendritic appendage of a geniculate interneuron. [file Image_2.jpg]

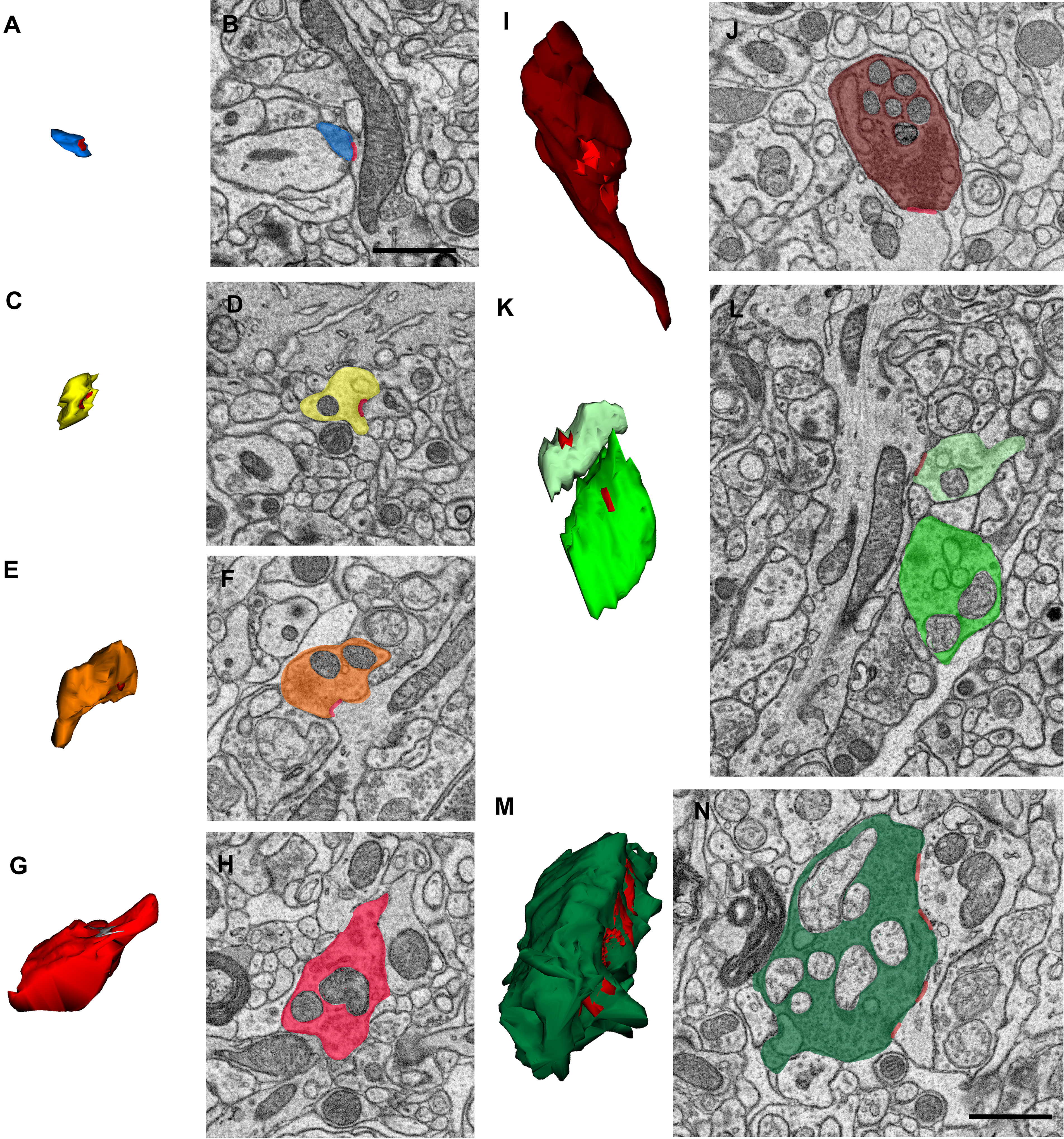

Supplement: Supplementary Figure 3 — Geniculate input boutons display a large range of sizes. Reconstructions (A,C,E,G,I,K,M) and a representative image of the section for each reconstruction (B,D,F,H,J,L,N, respectively) are paired in adjacent columns. The boutons that originate from retinal ganglion cells are identified by their unique mitochondrial morphology (panels K–N): the mitochondria appear pale or with light contrast, primarily owing to larger cristae and the cytoplasm that is lightly electron dense. While a wide range of bouton sizes are found, retinal boutons provide the largest-sized boutons in the geniculate circuitry. The boutons that contain no (panels A,B) or dark mitochondria (panels C–J) also represent a very wide range and include corticothalamic, brainstem, interneuron, and thalamic reticular nucleus inputs. Notice that the largest-sized non-retinal boutons can be within the size range of the largest retinal terminals. Scale bars in (B,N) = 500 nm, and apply to all panels. [file Image_3.jpg]
